# A survey of protein interactions and posttranslational modifications that influence the polyglutamine diseases

**DOI:** 10.3389/fnmol.2022.974167

**Published:** 2022-09-14

**Authors:** Sean L. Johnson, Wei-Ling Tsou, Matthew V. Prifti, Autumn L. Harris, Sokol V. Todi

**Affiliations:** ^1^Department of Pharmacology, Wayne State University, Detroit, MI, United States; ^2^Maximizing Access to Research Careers (MARC) Program, Wayne State University, Detroit, MI, United States; ^3^Department of Neurology, Wayne State University, Detroit, MI, United States

**Keywords:** aging, ataxia, hereditary diseases, misfolding and aggregation, neurodegenerative diseases, protein quality control

## Abstract

The presence and aggregation of misfolded proteins has deleterious effects in the nervous system. Among the various diseases caused by misfolded proteins is the family of the polyglutamine (polyQ) disorders. This family comprises nine members, all stemming from the same mutation—the abnormal elongation of a polyQ repeat in nine different proteins—which causes protein misfolding and aggregation, cellular dysfunction and disease. While it is the same type of mutation that causes them, each disease is distinct: it is influenced by regions and domains that surround the polyQ repeat; by proteins with which they interact; and by posttranslational modifications they receive. Here, we overview the role of non-polyQ regions that control the pathogenicity of the expanded polyQ repeat. We begin by introducing each polyQ disease, the genes affected, and the symptoms experienced by patients. Subsequently, we provide a survey of protein-protein interactions and posttranslational modifications that regulate polyQ toxicity. We conclude by discussing shared processes and pathways that bring some of the polyQ diseases together and may serve as common therapeutic entry points for this family of incurable disorders.

## Introduction

**FIGURE 1:**
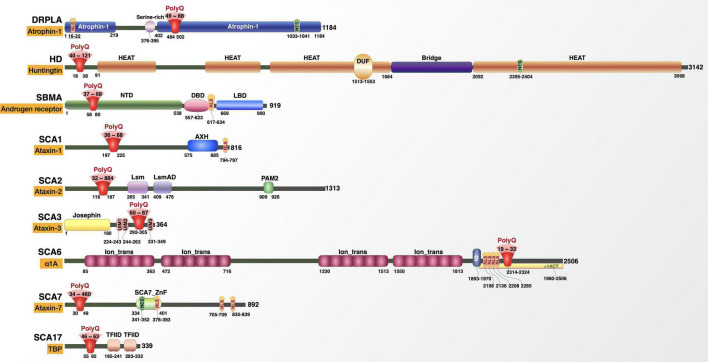
The polyglutamine disease proteins. Details on each protein’s domains, regions, posttranslational modifications, and interactions are found in Figures 2–10 and in the main text.

The polyglutamine (polyQ) disease family comprises nine inherited neurodegenerative disorders that are caused by the anomalous expansion of a CAG triplet repeat in the protein-coding region of each disease gene (Figure 1 and Table 1; Todi et al., 2007; Paulson et al., 2017; Lieberman et al., 2019). The CAG expansion encodes a prolonged polyQ tract in each disease protein that causes a host of shared features: slowly progressive neurodegeneration that typically manifests in the adult years of life, inverse correlation between the length of the polyQ expansion and age-of-onset, and autosomal dominant inheritance pattern that is shared by all but one of the diseases (Todi et al., 2007; Paulson et al., 2017; Lieberman et al., 2019).

**TABLE 1:** List of abbreviations.

Abbreviation	Definition
α1ACT	α1A subunit of human voltage-gated CaV2.1 (P/Q-type) Ca^2 +^ channel COOH-terminus
A2BP1	Ataxin-2-binding protein 1
Ada	Adenosine deaminase
AIS	Androgen-insensitivity syndrome
ALS	Amyotrophic lateral sclerosis
APC/C	Anaphase promoting complex/cyclosome
ARE	Androgen-responsive element
ATXN	Ataxin
ATXN1L	Ataxin-1-like protein
AXH	ATXN1/HBP1 domain
BDNF	Brain-derived neurotrophic factor
CaM	Calmodulin
CaMKII	Ca^2 +^/calmodulin-dependent protein kinase II
CAP	Cbl-associated protein
CHIP	C-terminus of Hsc70-interacting protein
CIC	Capicua
CNS	Central nervous system
CREB	cAMP-responsive element-binding protein
Crx	Cone-rod homeobox protein
DBD	DNA-binding domain
DHT	Dihydrotestosterone
DRPLA	Dentatorubral-pallidoluysian atrophy
DUB	Deubiquitinating enzyme
DUF	Domain of unknown function
ER	Endoplasmic reticulum
ERAD	ER-associated degradation
ETO/MTG8	Eight-twenty-one protein/Myeloid translocation gene on 8q22
Eve	Even-skipped
FOX-2	RNA-binding Fox-1 homolog 2
FQNLF	N-terminal FXXLF motif
Gcn5	General control non-depressible 5
GSK3β	Glycogen synthase kinase 3β
HAT	Histone deacetyltransferase
HD	Huntington’s disease
HDAC1	Histone deactyltransferase 1
HEAT	Huntingtin, elongation factor 3, A subunit of protein phosphatase 2A, TOR1
HIP1	Huntingtin-interacting protein 1
HIP14	Huntingtin-interacting protein 14
HIPPI	HIP1-protein interactor
Hsc70-4	Heat shock cognate 70-4
Hsp	Heat-shock protein
Htt	Huntingtin
Ion_trans	Ion transport
IP_3_R1	Type 1 inositol 1,4,5-triphosphate receptor
IQ	Isoleucine-glutamine motif
IRES	Internal ribosomal entry site
LANP	Leucine-rich acidic nuclear protein
LBD	Ligand-binding domain
LC3C	Microtubule-associated protein 1A/1B-light chain 3C
Lsm	Like Sm (Sm defined below)
LsmAD	LSM-associated domain
MANF	Mesencephalic astrocyte-derived neurotrophic factor
MyoD	Myogenic differentiation 1
NES	Nuclear export signal
NFY	Transcription factor nuclear factor-Y
NI	Nuclear inclusion
NLK	Nemo-like kinase
NLS	Nuclear localization signal
NMDA	N-methyl-D-aspartate receptor
NRSE	Neuron-restrictive silencer element
NTD	N-terminal domain
PAM2	Poly(A)-binding protein-interacting motif 2
PML-II	Promyelocytic leukemia protein isoform II
PolyQ	Polyglutamine
PPA2	Inorganic pyrophosphatase 2
PRMT6	Protein arginine methyltransferase 6
Prpf19	Pre-mRNA processing factor 19
PSD-95	Postsynaptic density protein 95 kDa
Rb	Retinoblastoma protein
RBM17	RNA-binding motif protein 17
RBP	Rab3-interacting molecule (RIM)-binding protein
RBP-J/Su(H)	Recombination signal-binding protein for immunoglobulin kappa J region
RE	Arginine-glutamic acid dipeptide repeat
REST	Repressor element-1 silencing transcription factor
RGS8	Regulator of G-protein signaling 8
RIM1/2	Rab-interacting molecules 1 and 2
RORα	Tip60/retinoic acid orphan related receptor alpha
sALS	Sporadic ALS
SAGA	Spt-Ada-Gcn5 acetyltransferase complex
SBMA	Spinal and bulbar muscular atrophy
SCA	Spinocerebellar ataxia
SH3P12/SORBS1	Sorbin and SH3 domain-containing protein 1
Sm	RNA-binding motif
SMA	Spinal muscular atrophy
SNP	Single-nucleotide polymorphism
Sp1	Specificity protein1 transcription factor
SR	Serine-rich
SUMO	Small ubiquitin-like modifier
Syt	Synaptotagmin
TAF	Transcription initiation factor
TBP	TATA-box binding protein
TDP-43	TAR DNA-binding protein of 43 kDa
TAFIID	Transcription factor II D
TAFII130/TAF4	TATA-box-binding protein associated factor 4
TRPC3	Transient receptor potential channel 3
t-SNARE	Target-localized SNARE (soluble NSF attachment protein receptor); NSF: N-ethyl-maleimide-sensitive protein
U2AF65	U2 snRNP auxiliary factor 65 kDa
Ubs	Ubiquitin-binding site
UIM	Ubiquitin-interacting motifs
UPS	Ubiquitin-proteasome system
USP	Ubiquitin-specific protease
VCP	Valosin-containing protein
XBP1	X-box-binding protein 1
ZnF	Zinc-finger domain

PolyQ diseases include dentatorubral-pallidoluysian atrophy (DRPLA), Huntington’s disease (HD), spinal and bulbar muscular atrophy (SBMA, also known as Kennedy’s disease), and spinocerebellar ataxias (SCAs) 1, 2, 3, 6, 7, and 17. While each disorder shares the same underlying genetic cause and characteristics described above (other than SBMA, which is X-linked), they are clinically distinct. Their disease proteins are widely expressed in the body and the central nervous system (CNS), but each disease features protein accumulation and degeneration in particular CNS regions that are associated with unique sets of symptoms (Todi et al., 2007; Paulson et al., 2017; Lieberman et al., 2019).

This review highlights advancements in the understanding of polyQ disorders and what differentiates the members of the polyQ family from one another; it is a survey of factors outside of the polyQ tract expansion that play a role in the toxicity of each disease protein. For each polyQ protein, there is a network of protein-protein interactions and domains that exist outside of the repeat that help to establish their individual “context” of pathogenesis. Additional influence arises from posttranslational modifications that have similar or divergent effects on different polyQ disease proteins. This “protein context” is critical in our understanding of these diseases and paints a comprehensive picture of interactions that can be targeted as potential therapeutic entry points. (Additional, contributing factors in polyQ disorders also exist outside the realm of the disease proteins themselves, including RNA-based toxicity and unconventional translation (Zu et al., 2011; Cleary et al., 2018; Guo et al., 2022); those mechanisms are outside the scope of this review).

In the following sections, we introduce each member of the family of polyQ diseases and then highlight domains and protein-protein interactions important for the context of each disease.

## PolyQ diseases and protein context

### Dentatorubral-pallidoluysian atrophy

#### The disease and its symptoms

DRPLA is a progressive ataxia characterized by movement, cognitive, and emotional abnormalities (Smith et al., 1958; Naito and Oyanagi, 1982; Ross et al., 1997; Nucifora et al., 2003; Todi et al., 2007; Lieberman et al., 2019). Additional manifestations vary depending on the age-of-onset, with patients who develop the disease before the age of 20 years typically experiencing epilepsy, myoclonus, and progressive intellectual deterioration, whereas individuals with onset after the age of 20 years exhibit cerebellar ataxia, choreoathetosis, and dementia with clinical similarity to HD. Repeat-expansion occurs in the *ATN1* gene located on chromosome 12p, which encodes atrophin-1, a transcriptional corepressor whose function is not well known (Li et al., 1993; Koide et al., 1994; Nagafuchi et al., 1994; Ikeuchi et al., 1995; Komure et al., 1995; Nucifora et al., 2003; Todi et al., 2007; Lieberman et al., 2019). The normal CAG tract of this gene ranges from 3 to 38 repeats with a pathogenic threshold beginning at expansions of 49 (Nucifora et al., 2003; Todi et al., 2007; Lieberman et al., 2019).

**FIGURE 2:**
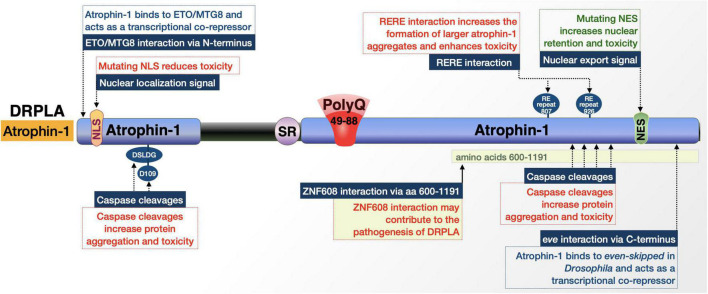
Graphic representation of the DRPLA protein, its domains, interactions, and modifications. Details are in the main text.

Atrophin-1 (Figure 2) is a hydrophilic, 1184 amino acid-long protein with several simple repetitive motifs including a serine-rich region, the variable polyQ tract, a polyproline tract, and a region of alternating acidic and basic residues (Yazawa et al., 1995; Margolis and Ross, 2001; Nucifora et al., 2003; Todi et al., 2007; Lieberman et al., 2019). It also possesses a nuclear-localization signal (NLS) in its N-terminus and a nuclear-export signal (NES) in its C-terminus (Miyashita et al., 1997; Okamura-Oho et al., 1999; Nucifora et al., 2003; Todi et al., 2007; Lieberman et al., 2019). With these intrinsic signaling motifs, atrophin-1 localizes to both the cytoplasm and the nucleus of neurons and appears in both neuronal nuclear inclusions (NI) as well as cytoplasmic polyQ aggregates, neither of which necessarily correlates directly with toxicity (Yazawa et al., 1995, 1997; Knight et al., 1997; Becher and Ross, 1998; Sato et al., 1999; Nucifora et al., 2003; Todi et al., 2007; Lieberman et al., 2019).

While it is expressed widely in the brain of both normal and affected individuals (Nagafuchi et al., 1994; Yazawa et al., 1997; Margolis and Ross, 2001), the primary targets for degeneration upon atrophin-1 expansion are the dentate nucleus of the cerebellum, red nucleus, globus pallidus, and the subthalamic nucleus. This clinical range of pathological presentations among patients with essentially the same mutation hints at additional genetic and environmental factors playing a role in DPRLA pathogenesis (Nucifora et al., 2003; Todi et al., 2007; Lieberman et al., 2019), some of which we discuss next.

#### The role of atrophin-1 localization signals and proteolytic processing in DRPLA

As is the case with other disease proteins that we survey here, atrophin-1 is subjected to cleavage events and has additional protein-protein interactions that exacerbate or suppress its toxicity in various models of DRPLA. The following paragraphs capture this information and are representative of the textual organization of the remainder of this review article.

##### Proteolytic processing and sub-cellular localization

Atrophin-1 is reported to have multiple interactions with caspases that cleave it and yield fragments that may alter its regulatory mechanisms upon polyQ expansion and increase cellular toxicity (Figure 2; Ellerby et al., 1999a; Nucifora et al., 2003; Suzuki et al., 2010). Many of these cleavages and their purported toxic effects center around separating the atrophin-1 N-terminal NLS or C-terminal NES from the remainder of the protein and its expanded polyQ (Nucifora et al., 2003). Caspase cleavage separating the NLS from the rest of the protein results in a C-terminal fragment that is more prone to cytoplasmic accumulation and has been observed in postmortem DRPLA patient tissue. These events, addressed in the following paragraphs, highlight caspase activity as critical for expanded atrophin-1’s cellular toxicity, altered nuclear localization, and aggregation. (Nucifora et al., 2003; Suzuki et al., 2010).

Cleavage by caspase 3 at D109 [N-terminal 106 (DSLDG)110] in an expanded state separates the NLS from the rest of the protein and produces a pro-apoptotic fragment that may contribute to aggregation, but does not affect atrophin-1 localization (Miyashita et al., 1997; Ellerby et al., 1999a). Mutation of this site to prevent cleavage blocks the creation of this fragment and markedly reduces cellular toxicity; however, whether this caspase-3-dependent effect occurs *in vivo* has not been confirmed (Ellerby et al., 1999a).

As mentioned above, atrophin-1 contains both NLS and NES that regulate its localization within the cell. The NES seems to be important for DRPLA; fragments generated by unknown caspases that separate the NES from the polyQ and NLS show increased nuclear retention and toxicity (Nucifora et al., 2003). DRPLA patients and transgenic model mice have nuclear accumulation of atrophin-1, particularly a ∼120 kDa fragment that would be representative of a cleavage product. Mutation of full-length, polyQ-expanded atrophin-1 to render the NES non-functional in cultured Neuro2a cells resulted in increased nuclear localization of the protein and an increase in cellular toxicity. Furthermore, mutating the NLS in the fragment lacking an NES shifted atrophin-1 more to the cytoplasm and reduced cellular toxicity. This fragment does not appear to result from the activity of caspase 2, 3, 6, 7, or 8; the exact enzyme remains to be identified (Nucifora et al., 2003). These studies indicate that the NLS and NES both play critical roles in polyQ-expanded atrophin-1 toxicity and that various caspase cleavages modulate nuclear localization, aggregate formation, and toxicity in DRPLA.

##### Protein-protein interactions

Atrophin-1 has direct interactions that may impact toxicity. One is through one of its two arginine-glutamic acid (RE) dipeptide repeats (Yanagisawa et al., 2000). The interaction is with the product of the RE repeat-encoding gene, designated as *RERE*, which shares 67% sequence homology with atrophin-1. The *RERE-*encoded protein, RERE, and atrophin-1 heterodimerize and colocalize in a speckled pattern in the nucleus; this interaction is strengthened when the atrophin-1 polyQ is pathologically expanded. The enhanced interaction results in the formation of larger atrophin-1 aggregates, which may further exacerbate toxicity (Yanagisawa et al., 2000).

Additional factors that may have physiological consequences in DRPLA include atrophin-1’s interaction with the transcriptional regulator ETO/MTG8 (Wood et al., 2000); *Drosophila* atrophin-1 homolog’s interaction with *even-skipped* (Zhang et al., 2002)*;* and the interaction of the *Drosophila* ZNF608/ZNF609 homolog, *brakeless* with atrophin-1, which regulates atrophin-1’s function as a transcriptional corepressor (Haecker et al., 2007). The implications of these interactions for DRPLA are not clear. Continuing research will undoubtedly yield further details on the overall protein context of DRPLA.

### Huntington’s disease

#### The disease and its symptoms

HD is the most common polyQ disorder in the United States (Li and Li, 2004; Todi et al., 2007; Jones and Hughes, 2011; Ghosh and Tabrizi, 2018). While its first descriptions were in 1872 by George Huntington, the actual mutation was not discovered until 1993 (The Huntington’s Disease Collaborative Research Group, 1993). HD is a single-gene disorder with autosomal dominant transmission. Motor, psychiatric, and cognitive symptoms begin in middle age and progress over the next 10–15 years, leading to patient death. Patients often experience psychiatric and cognitive symptoms, along with subtle motor deficits, for years before official disease onset, which correlates with the length of the CAG repeat. The areas of the brain primarily impacted in this disease are the striatum and the deep layers of the cortex this includes atrophy of the cerebral cortex and the subcortical white matter and, as the disease progresses, spreads to other brain regions like the hypothalamus and hippocampus (Li and Li, 2004; Todi et al., 2007; Jones and Hughes, 2011; Ghosh and Tabrizi, 2018). Degenerative losses include up to 95% of the GABAergic medium spiny neurons that project to the globus pallidus and substantia nigra (Ghosh and Tabrizi, 2018).

The polyQ expansion in huntingtin originates from the *HTT* gene on the short arm of chromosome 4 (Ghosh and Tabrizi, 2018). A non-HD allele has 6 to 35 CAG repeats; the upper limit of the non-pathogenic threshold is 27–35 and is referred to as intermediate allele. Beyond the intermediate span is a range from 36 to 39 repeats that is not fully penetrant, and 40 to as many as 121 repeats represent the fully penetrant mutations (Li and Li, 2004; Todi et al., 2007; Jones and Hughes, 2011; Ghosh and Tabrizi, 2018). As is the case with most polyQ disorders, *HTT* expansions exhibit anticipation; i.e., they tend to get longer as they are passed from one generation to the next and lead to progressively earlier onset, since longer repeats are inversely correlated with the age-of-onset.

**FIGURE 3:**
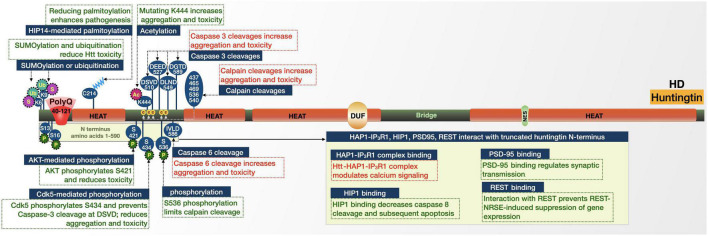
Graphic representation of the HD protein, its domains, interactions, and modifications. Details are in the main text.

The 350 kDa huntingtin protein has an N-terminal polyQ tract that begins at residue 18 (Figure 3; Li and Li, 2004; Todi et al., 2007; Jones and Hughes, 2011; Ghosh and Tabrizi, 2018). This repeat is followed by two polyproline repeats of 11 and 10 residues each, making up the external loop structure of the N-terminus. The remainder mostly comprises a series of four sections of HEAT (huntingtin, elongation factor 3, A subunit of protein phosphatase 2A, TOR1) repeats that are found in a variety of proteins involved in intracellular transport and chromosomal segregation; they mediate huntingtin’s protein-protein interactions (Li and Li, 2004; Jones and Hughes, 2011; Ghosh and Tabrizi, 2018; Guo et al., 2018). The HEAT repeats can be sub-categorized into N- and C-terminal regions separated by a bridge domain (Guo et al., 2018). The N-terminal assembly contains 21 HEAT repeats spread across the first three HEAT repeat sections and spans residues 91-1684. The C-terminal domain possesses the fourth HEAT (residues 2092-3098), itself comprising 12 HEAT repeats (Guo et al., 2018). Although no classical NLS has been reported, huntingtin does have an NES near the C-terminus (Xia et al., 2003; Jones and Hughes, 2011) and certain types of phosphorylation and proteolytic processing alter its sub-cellular localization (Schilling et al., 2006).

Huntingtin is present in nearly all tissues. Subcellularly, it is found in the nucleus and also associates with the endoplasmic reticulum (ER), Golgi complex, synaptic vesicles, and mitochondria. Many of huntingtin’s interactions with other proteins occur through its N-terminal region (amino acids 1-588) and are linked to either gene transcription or intracellular signaling, trafficking, endocytosis, and metabolism (Li and Li, 2004; Todi et al., 2007; Jones and Hughes, 2011). According to expansive research and various unbiased and targeted assays, huntingtin has numerous interacting partners, (Li and Li, 2004; Shirasaki et al., 2012; Langfelder et al., 2016; Vijayvargia et al., 2016; Alanis-Lobato et al., 2017; Guo et al., 2018; Wanker et al., 2019). The large number of huntingtin-binding proteins—alongside evidence that it is implicated in several cellular processes—suggest that the HD protein is a molecular scaffold. Whatever its exact functions, huntingtin is an essential protein, indicating a loss-of-function mechanism of neurodegeneration; however, there is also evidence that the expanded polyQ is linked to a toxic gain-of-function that plays a role in HD pathogenesis, adding to the complexity of this disease.

#### How non-polyQ regions of huntingtin and their posttranslational modifications influence HD

##### Proteolytic processing

Huntingtin is processed by proteases, resulting in N-terminal fragments (Goffredo et al., 2002; Lunkes et al., 2002; Li and Li, 2004; Benn et al., 2005; Jones and Hughes, 2011; Ghosh and Tabrizi, 2018). Several sites within huntingtin, primarily its first 600 amino acids, are caspase cleavage sites (Kim et al., 2001; Gafni and Ellerby, 2002; Lunkes et al., 2002; Wellington et al., 2002). These fragments tend to appear in higher concentrations in the nucleus compared with the full-length protein and are more susceptible to accumulation and aggregation following polyQ expansion (Jones and Hughes, 2011). Whether these aggregates are harmful or protective is not entirely settled, but may involve several proteins and pathways that are discussed further below. In HD mouse models, modification of huntingtin to prevent caspase-6 cleavage at amino acid 586 helped maintain normal neuronal function and eliminated striatal neurodegeneration and motor dysfunction (Figure 3; Graham et al., 2006; Truant et al., 2007). In addition to mutant huntingtin fragments produced by proteolysis, incomplete splicing of mutant *HTT* RNA generates a short protein consisting of exon 1 (Neueder et al., 2017). This splice-form is highly pathogenic and has been observed in mouse HD models and in postmortem HD patient brain tissue. Mutant huntingtin RNA fragments have also been reported to sequester splicing machinery and deregulate splicing in cell and human brain models of HD (Lin et al., 2016; Schilling et al., 2019).

##### Posttranslational modifications

Huntingtin has sites for phosphorylation, SUMOylation, acetylation, ubiquitination, and palmitoylation. Each modification can alter the toxicity of the disease-causing variant. Phosphorylation generally appears to protect cells from the toxic effects of mutated huntingtin. Huntingtin is phosphorylated by Cdk5, which reduces the cleavage of huntingtin by caspase-3 at amino acid 513. Phosphorylation and the resulting protection from cleavage reduces aggregation and toxicity in cell models of HD. However, in HD there are lower levels of Cdk5, which leads to reduced phosphorylation of huntingtin; thus, afforded protections from cleavage and subsequent aggregation and toxicity are reduced (Luo et al., 2005; Schilling et al., 2006). Given the more toxic nature of N-terminal huntingtin fragments and the evidence of natural generation and accumulation of N-terminal expanded huntingtin fragments in HD mouse brains (Davies et al., 1997; Hackam et al., 1998; Reddy et al., 1998; Hodgson et al., 1999; Schilling et al., 1999; Zhou et al., 2003; Li and Li, 2004; Jones and Hughes, 2011), it stands to reason that cleavage at these sites plays a role in enhancing toxicity in HD.

Similar to the effect by Cdk5, AKT-mediated phosphorylation of S421 of expanded huntingtin relieved neurotoxicity in cultured cells (Humbert et al., 2002; Gauthier et al., 2004; Pardo et al., 2006; Metzler et al., 2007, 2010; Colin et al., 2008). Comparable amelioration was observed upon phosphorylation of expanded huntingtin at several sites (Schilling et al., 2006; Gu et al., 2009; Thompson et al., 2009): N-terminal phosphorylation targets huntingtin for degradation (Gu et al., 2009; Thompson et al., 2009); S536 phosphorylation limits its calpain cleavage (Schilling et al., 2006); S421 phosphorylation facilitates intracellular neuronal transport, and may also limit toxicity by increasing proteasomal turnover of mutant huntingtin (Gauthier et al., 2004; Colin et al., 2008; Kratter et al., 2016).

Acetylation and palmitoylation are also protective in HD. Mutations to prevent acetylation of huntingtin at K444 resulted in increased accumulation and neurodegeneration in cultured neurons and mouse brains (Jeong et al., 2009). Similar results were observed upon mutation of huntingtin’s C214 palmitoylation site (Yanai et al., 2006). C214 palmitoylation is performed by huntingtin-interacting protein 14 (HIP14). The association between huntingtin and HIP14 is reduced by polyQ expansion, resulting in reduced palmitoylation and increased inclusion formation, a potential contributor to HD (Yanai et al., 2006).

Huntingtin’s K6 and K9 residues are sites of post-translational modification with influence on two fronts: SUMOylation at both sites reduced the ability of an expanded N-terminal huntingtin fragment to aggregate and promoted its capacity to repress transcription in a cell model (Steffan et al., 2004). However, in a *Drosophila* model expressing exon 1 of huntingtin, SUMOylation at the same sites enhanced neurodegeneration, while ubiquitination was protective (Steffan et al., 2004). These seemingly conflicting results point to a role for SUMOylation in HD pathogenesis that requires further elucidation.

##### Protein-protein interactions

Huntingtin has several direct interactions that influence its toxicity, particularly with proteins involved in intracellular trafficking and signaling. Huntingtin-associated protein 1 (HAP1), interacts with both huntingtin and the type 1 inositol 1,4,5-trisphosphate receptor (IP_3_R1) to form a ternary complex (Li et al., 1995; Tang et al., 2003). The presence of expanded huntingtin in this complex enhances the sensitivity of IP_3_R1 to inositol 1,4,5-trisphosphate and provides an explanation for the changes in Ca^2+^ signaling observed in HD patients (Tang et al., 2003).

Another huntingtin-associated protein, huntingtin-interacting protein (HIP1), has decreased binding to polyQ-expanded huntingtin compared to wild-type (Kalchman et al., 1997; Wanker et al., 1997; Gervais et al., 2002). The decreased binding may increase the amount of free HIP1, which can then associate with HIP1-protein interactor (HIPPI) and induce caspase-8-mediated apoptosis (Gervais et al., 2002). Mutant huntingtin also has a decreased interaction with postsynaptic density protein 95 (PSD-95), a scaffold that regulates clustering and activation of postsynaptic membrane receptors (Sun et al., 2001). HD patients release more PSD-95 than unaffected individuals, which results in over-activation of N-methyl-D-aspartate (NMDA) receptors and associated abnormal synaptic transmission, which were validated in HD mice (Cepeda et al., 2001; Zeron et al., 2002).

The final set of huntingtin interactions that we discuss here are transcriptional-related. Wild-type huntingtin interacts with repressor element-1 transcription factor (REST) in the cytoplasm (Zuccato et al., 2003, 2008). REST, in association with the neuron restrictive silencer element (NRSE), regulates neuronal gene transcription, including brain-derived neurotrophic factor (BDNF). The interaction between huntingtin and REST-NRSE is weaker when its polyQ repeat is expanded. Consequently, while wild-type huntingtin may interact with REST-NRSE in the cytoplasm to prevent entry into the nucleus and reduce the REST-NRSE-induced suppression of gene expression, mutant huntingtin limits this effect, subsequently inhibiting BDNF and other genes (Zuccato et al., 2003, 2008). PolyQ-expanded huntingtin also inhibits the function of several transcriptional coactivators, including p53 and CBP (Steffan et al., 2000, 2001).

Based on the work conducted thus far, a variety of losses-of- and gains-of-functions that are specific to the HD protein lead to symptomatology that separates this disease from the others in its family.

### Spinal and bulbar muscular atrophy

#### The disease and its symptoms

**FIGURE 4:**
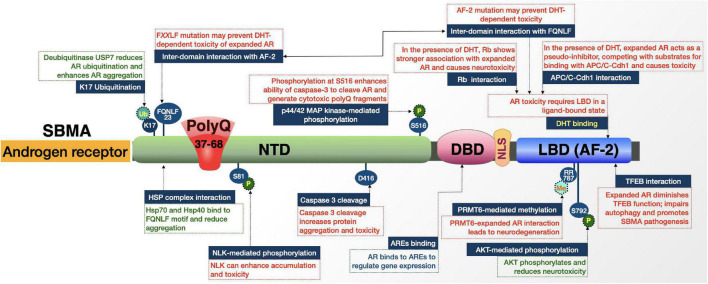
Graphic representation of the SBMA protein, its domains, interactions, and modifications. Details are in the main text.

SBMA is a late-onset neuromuscular disorder caused by the expansion of a polymorphic polyQ repeat in the androgen receptor (AR) protein (Figure 4). In 1991, SBMA was the first identified polyQ disease; it has several features that make it unique among its family of disorders (La Spada et al., 1991). For example, it is the only X-linked polyQ disease and thus the only one not inherited autosomal dominantly (Todi et al., 2007; Beitel et al., 2013; Cortes and La Spada, 2018). The existence of the polyQ expansion in the AR protein also means that SBMA is the sole ligand-dependent polyQ disorder; it shares its causative protein with other diseases including androgen-insensitivity syndrome (AIS) and prostate cancer (Gottlieb et al., 2004, 2012; Beitel et al., 2013; Cortes and La Spada, 2018). This overlap in disease proteins means that men with SBMA also experience mild signs of AIS-like gynecomastia and some degree of infertility, in addition to the late-onset and progressive symptoms of neurodegeneration, dysarthria, and dysphagia that are shared among most polyQ disorders. Some of the AR-specific effects in both SBMA and AIS are suspected to result from AR loss-of-function; however, AIS symptoms in SBMA and absence of neuromuscular phenotypes in AIS indicate that AR polyQ expansion also leads to gain-of-function that is selectively harmful to motor neurons. While the X-linked nature of the disease means that full penetrance only occurs in males, women who are heterozygous for polyQ-expanded AR may experience subclinical effects like muscle cramps and electrophysiological abnormalities; but, the low circulating levels of androgens protects them from degeneration (Todi et al., 2007; Beitel et al., 2013; Cortes and La Spada, 2018).

For males, SBMA is detrimental to skeletal muscle and causes progressive muscle cramps, weakness and wasting, and twitching. These symptoms can result in SBMA patients being initially misdiagnosed with disorders such as amyotrophic lateral sclerosis (ALS) or autosomal recessive spinal muscular atrophy (SMA). Distinguishing among these diagnoses is critically important for the patient because, while the mean survival time with ALS is only a few years, the life expectancy for SBMA patients is normal or only minimally reduced (Beitel et al., 2013; Cortes and La Spada, 2018).

Pathologically, SBMA is characterized by the loss of lower motor neurons of the brainstem and anterior horn of the spinal cord, and to a lesser extent of the sensory neurons of the dorsal root ganglia (Todi et al., 2007; Beitel et al., 2013; Cortes and La Spada, 2018). This degeneration leads to weakness and atrophy of the bulbar, facial, and limb muscles (Beitel et al., 2013; Cortes and La Spada, 2018). It has been proposed that the toxicity of polyQ-expanded AR originates in skeletal muscle and results in secondary motor neuron degeneration (Beitel et al., 2013; Giorgetti et al., 2016; Cortes and La Spada, 2018).

As noted above, polyQ expansion in SBMA occurs in AR, the male steroid receptor encoded by the *AR* gene on the short arm of the X chromosome. This gene contains 8 coding exons consisting of three functional domains that are shared among the super-family of steroid-binding transcription factors. The polymorphic CAG repeat is in the first exon and normally possesses 5–34 repeats, but expands to 37 or more repeats in pathogenic alleles; shorter lengths are associated with increased risk of prostate cancer (Beitel et al., 2013; Cortes and La Spada, 2018). [The AR sequence actually has 3 polyQ-encoding repeat tracts, but the first one (exon 1) is by far the longest (Cortes and La Spada, 2018)].

The normal biology of AR is amply studied and well understood, allowing for greater understanding of the relationship between native AR function and polyQ-expanded AR dysfunction. AR is a ligand-activated transcription factor and member of the nuclear receptor super-family. AR consists of an N-terminal domain (NTD) that modulates transcriptional activation, a central deoxyribonucleic acid (DNA)-binding domain (DBD) that binds androgen-responsive elements (AREs), and a C-terminal ligand-binding domain (LBD). It also has several subdomains that are involved in nuclear localization, dimerization, and interaction with heat shock proteins (HSPs), co-activators and other proteins (Beitel et al., 2013; Cortes and La Spada, 2018).

In the absence of androgens, AR resides in the cytoplasm in a heteromeric, inactive complex with HSPs. Upon binding of its ligand, testosterone, or its metabolite 5α-dihydrotestosterone (DHT), AR undergoes conformational changes that promote its dissociation from the HSP complex and exposes the AR NLS, DBD, and dimerization domains. This exposure promotes dimerization and nuclear translocation, allowing AR to bind AREs in promoter regions and recruit transcriptional co-activators for androgen-sensitive genes. Wild-type AR also interacts with proteins involved in RNA-splicing, protein translation, proteasome/protein ubiquitination, transcription, and male sexual differentiation and development (Beitel et al., 2013; Cortes and La Spada, 2018). Each of these normal mechanisms has been implicated in SBMA pathogenesis upon polyQ expansion.

#### Androgen receptor domains, interactions, and modifications that regulate SBMA

##### Proteolytic processing and protein-protein interactions

Like polyQ diseases discussed above and additional ones that follow, AR is subjected to proteolytic cleavage (Ellerby et al., 1999b). According to cultured cell experiments, polyQ-expanded AR can be cleaved by caspase-3, leading to the induction of apoptosis. Prevention of this cleavage event reduced the formation of AR aggregates and precluded apoptosis in this system (Ellerby et al., 1999b).

As noted above, AR functions in a ligand-dependent manner. Many of the protein-protein interactions and domains of influence in expanded-AR toxicity are possible when AR-LBD is in a ligand-bound state (Figure 4). The binding of a ligand to polyQ-expanded AR promotes conformational changes, ligand-dependent unfolding, and nuclear translocation. All these steps are critical in SBMA pathogenesis and impact the AR interactome (Lieberman et al., 2014).

In the presence of DHT, retinoblastoma (Rb) protein shows a stronger association with polyQ-expanded AR compared to wild-type AR (Suzuki et al., 2009). Rb normally acts as neuroprotective factor that recruits histone deacetyltransferase 1 (HDAC1) in the co-repression of E2F1, a transcription factor whose target genes regulate apoptosis, development, and differentiation (Suzuki et al., 2009). The enhanced association between Rb and polyQ-expanded AR suppresses Rb’s ability to recruit HDAC1 and leads to aberrant E2F1 transcriptional activation.

Another ligand-dependent AR interaction involves DHT-induced cell cycle arrest; polyQ expansion alters the function of AR in cell cycle regulation (Suzuki et al., 2009). Under both normal and polyQ-expanded conditions, AR interacts with the anaphase promoting complex/cyclosome (APC/C) and its adaptor, Cdh1, in a DHT-dependent manner (Bott et al., 2016). Cultured cell studies revealed the possibility that expanded AR acts as a pseudo-inhibitor, competing with substrates for binding with APC/C-Cdh1 through a toxic gain-of-function that causes abortive neuronal differentiation followed by mitotic re-entry (Bott et al., 2016).

The AF-2 domain of AR also plays a role in interactions that facilitate polyQ expansion-dependent toxicity. Within AR, the inter-domain interaction (N/C interaction) between the C-terminal AF-2 domain and N-terminal FXXLF motif (FQNLF) is important for AR aggregation and toxicity; while this is a normal step in wild-type AR metabolism, it is also thought to be a critical, early step in abnormal stabilization, aggregation, and toxicity of polyQ-expanded AR (Nedelsky et al., 2010; Orr et al., 2010; Zboray et al., 2015). This notion is supported by studies in cultured cells where ligand and mutations were introduced that prevent the interaction of FXXLF with AF-2 and subsequently abolished toxicity and aggregation of AR, rescuing primary motor neurons from ligand-induced toxicity. There was an additional suggestion that there are coactivators of this N/C interaction including GRIP1, F-src-1, and CBP that could further stabilize polyQ-expanded AR (Orr et al., 2010). The AF-2 domain is also the site of AR’s interaction with transcription factor EB (TFEB). AR normally functions as a co-activator of TFEB; however, polyQ expansion changes this interaction to an inhibitory one, causing TFEB dysregulation that leads to autophagic flux impairment observed in SBMA models (Cortes et al., 2014).

##### Posttranslational modifications

Various modifications impact the pathogenicity of polyQ-expanded AR. For example, the AF-2 domain interacts with protein arginine methyltransferase 6 (PRMT6) (Scaramuzzino et al., 2015). This association is enhanced by polyQ expansion and is a double-edged sword for SBMA due to the phosphorylation of AR by AKT and the role of PRMT6 on this modification. On the one hand, phosphorylation of polyQ-expanded AR by AKT reduces ligand binding and AR transactivation, protecting from neurodegeneration. On the other hand, PRMT6 methylates arginine residues at the AKT consensus sites, reducing AKT-dependent phosphorylation. Therefore, while phosphorylation by AKT is generally neuroprotective, enhanced interaction of PRMT6 with poly-Q-expanded AR, followed by increased arginine methylation, enhances neurodegeneration based on cell and *Drosophila* models of SBMA (Scaramuzzino et al., 2015).

AKT and PRMT6 are not the only posttranslational modifiers of mutant AR. Nemo-like kinase (NLK) can proliferate the accumulation and toxicity of polyQ-expanded AR through N-terminal binding and S81 phosphorylation, which aberrantly increases activation of various genes (Todd et al., 2015). In another set of posttranslational studies, phosphorylation at S516 *via* the p44/42 MAP kinase pathway induced cell death in an SBMA cell model (Lin et al., 2001; LaFevre-Bernt and Ellerby, 2003). This latter study suggests that phosphorylation enhances the ability of caspase-3 to cleave polyQ-expanded AR and generate toxic fragments (Lin et al., 2001; LaFevre-Bernt and Ellerby, 2003). Ligand-dependent hyperacetylation has also been reported in polyQ-expanded AR, compared to wild-type, and seems to be associated with toxicity in SBMA (Montie et al., 2011).

Finally, there is a differential interaction between wild-type and polyQ-expanded AR with ubiquitin-specific protease 7 (USP7) (Pluciennik et al., 2021). PolyQ-expanded AR preferentially associates with USP7. Ubiquitination at K17 of AR is a site of action for USP7 and increased interaction with expanded AR results in changes in the AR ubiquitination pattern that enhances its aggregation and toxicity. Based on studies with huntingtin and ataxin-3, USP7 may similarly impact polyQ disease proteins more generally (Pluciennik et al., 2021).

In sum, a combination of loss-of-function and gain-of-function properties, dictated by the context surrounding the polyQ repeat of AR, converge in a type of perfect storm that leads to cellular dysfunction and disease with clinical representations specific for this disorder.

### Spinocerebellar ataxia type 1

#### The disease and its symptoms

SCA1 was first described in 1993, when an unstable CAG repeat was identified in *ATXN1* (Orr et al., 1993). SCA1 is another adult-onset, progressive, inherited ataxia that presents with cognitive impairment, difficulty with speaking and swallowing, and eventual chronic lung infections and respiratory failure (Todi et al., 2007; Ju et al., 2014; Pérez Ortiz and Orr, 2018; Tejwani and Lim, 2020). Diagnosis is typically in the third or fourth decade of life, with the disease duration ranging from 10 to 30 years depending on the size of the repeat expansion. Symptoms typically begin with gait ataxia and progress to the point of wheelchair necessity within 15 years (Todi et al., 2007; Ju et al., 2014; Pérez Ortiz and Orr, 2018; Tejwani and Lim, 2020). Patients with SCA1 comprise approximately 6% of all autosomal dominant cerebellar ataxias, with large variances across ethnic groups (Tejwani and Lim, 2020). The primary pathological pathways of SCA1 are degeneration of the cerebellum and the brain stem, as well as atrophy of the ventral pons and middle cerebellar peduncles. There is major loss of cerebellar Purkinje cells and neurons of the inferior olivary nucleus, cortical, subcortical, and spinal structures (Todi et al., 2007; Ju et al., 2014; Pérez Ortiz and Orr, 2018; Tejwani and Lim, 2020).

**FIGURE 5:**
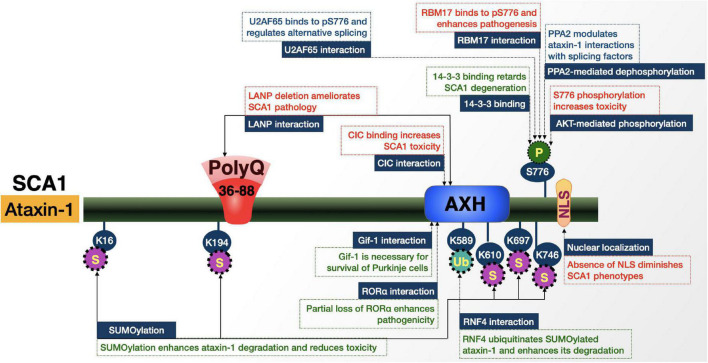
Graphic representation of the SCA1 protein, its domains, interactions, and modifications. Details are in the main text.

*ATXN1* is located on chromosome 6p and encodes the 87 kDa protein, ataxin-1 (Figure 5; Banfi et al., 1994; Todi et al., 2007; Tejwani and Lim, 2020). This protein is widely expressed throughout the CNS. Of the 9 exons of *ATXN1*, only exons 8 and 9 contain protein-coding sequences; exon 8 contains the CAG repeat. The repeat normally contains 4–36 uninterrupted triplets; alleles exist with > 21 repeats that also contain 1-3 CAT codons that translate to histidine (Todi et al., 2007; Ju et al., 2014; Pérez Ortiz and Orr, 2018; Tejwani and Lim, 2020). The interrupting histidine residues reduce aggregation of expanded ataxin-1 and are often lost in fully penetrant variants. With that said, uninterrupted CAG repeats of > 39 and interrupted CAG repeats of 43 or more result in the highly penetrant, late-onset, and progressive ataxia described above that additionally displays the typical correlation between polyQ tract length and disease severity. Tracts with 36–38 repeats have an intermediate effect that can result in ataxia without some of the other SCA1-specific features (Tejwani and Lim, 2020).

Ataxin-1 is involved in the regulation of transcription factors, although its functions are not all well understood (Mizutani et al., 2005; Todi et al., 2007; Pérez Ortiz and Orr, 2018; Tejwani and Lim, 2020). In addition to SCA1, ataxin-1 is also implicated in other neurodegenerative diseases. For example, expansions of 32 or more repeats enhance the risk of sporadic ALS (sALS). Additionally, a SNP (single-nucleotide polymorphism; rs179943) in an intronic region is associated with a higher risk of developing late-onset Alzheimer’s disease (Bertram et al., 2008; Tejwani and Lim, 2020).

#### Ataxin-1 is regulated by its AXH domain, cellular localization, and posttranslational modifications

##### Protein-protein interactions

SCA1 pathology does not depend solely on the polyQ tract of the ataxin-1 protein; several domains are essential for the induction of disease phenotypes even in the presence of an expanded polyQ, including the ataxin-1/HBP-1 (AXH) domain, its NLS, and phosphorylation at S776 (Figure 5; Todi et al., 2007; Ju et al., 2014; Pérez Ortiz and Orr, 2018; Tejwani and Lim, 2020). These regions function individually and in coordination with one another to shape the interactome of ataxin-1.

The AXH domain mediates various protein-protein interactions; as such, it is responsible for many of the interactions that impact toxicity upon polyQ expansion. Possibly the most important of these AXH-mediated interactions in SCA1 is expanded ataxin-1’s binding to the transcriptional repressor Capicua (CIC) (Lam et al., 2006; Bowman et al., 2007; Lim et al., 2008; Fryer et al., 2011; Kim et al., 2013; de Chiara et al., 2014; Lasagna-Reeves et al., 2015; Rousseaux et al., 2018; Tejwani and Lim, 2020). According to mouse studies, a majority of wild-type and expanded ataxin-1 assembles into large stable complexes with CIC (Lam et al., 2006). This assembly stabilizes ataxin-1 and allows for the induction of neurotoxicity; the direct interaction between CIC and expanded ataxin-1 alters CIC’s transcriptional activity (Lam et al., 2006). As further evidence of the important role of CIC in SCA1, mutating key amino acids to prevent CIC-ataxin-1 interaction also prevented toxicity in a Purkinje-cell specific SCA1 transgenic mouse model (Kim et al., 2013; Rousseaux et al., 2018), and haploinsufficiency of *Cic* reduced disease severity in SCA1 knock-in mice (Fryer et al., 2011). Ataxin-1’s incorporation into a CIC-containing protein complex appears critical for its stabilization, particularly in an expanded state, and confers toxicity through this mechanism as well as through the alteration of CIC transcriptional repression activity.

The AXH domain also mediates ataxin-1’s interaction with Gif-1. This transcription factor is necessary for the survival of Purkinje neurons and is destabilized by its interaction with ataxin-1. Destabilization enhances Gif-1 degradation *via* the proteasome, which contributes to Purkinje cell degeneration in SCA1 (Tsuda et al., 2005).

The AXH domain additionally facilitates ataxin-1’s interaction with Tip60/retinoic acid orphan related receptor alpha (RORα) (Serra et al., 2006; Gehrking et al., 2011) and the highly conserved paralog, ataxin-1-like (ATXN1L) (Bowman et al., 2007). These interactions involve incorporation into larger complexes that alter their stability and activity when the polyQ of ataxin-1 is expanded. Such changes can impact the levels of expanded ataxin-1 and further contribute to SCA1 (Serra et al., 2006; Bowman et al., 2007; Gehrking et al., 2011).

The final interaction of interest through the AXH is with leucine-rich acidic nuclear protein (LANP). This interaction occurs at both the AXH domain and the polyQ tract of ataxin-1 and is strengthened as the polyQ repeat increases (Matilla et al., 1997). Expanded ataxin-1 competes for LANP binding with the transcriptional repressor E4F (Cvetanovic et al., 2007); this gain-of-function, in conjunction with LANP expression pattern, may further underscore Purkinje cell-specific vulnerability observed in SCA1 (Opal et al., 2003; Cvetanovic et al., 2012). In fact, LANP reduction reversed molecular layer thinning in SCA1 knock-in mice (Cvetanovic et al., 2012).

Apart from direct protein-protein interactions, the AXH domain is also a player in the self-aggregation of ataxin-1. The isolated AXH domain forms β-plaques *in vitro*, while expanded ataxin-1 with its AXH domain replaced with a homologous sequence from the transcription factor HBP1, which is not known to aggregate, showed significantly reduced protein aggregation (de Chiara et al., 2005), further emphasizing the importance of the AXH domain in SCA1.

##### Sub-cellular localization

The C-terminal NLS of ataxin-1 is necessary for pathogenesis; mutation of the NLS essentially eliminated ataxin-1-based toxicity in an SCA1 transgenic mouse model (Klement et al., 1998). Near the C-terminal NLS is another important domain that influences expanded ataxin-1 toxicity, the 14-3-3 binding domain (around amino acids 774–778). Ataxin-1 binds 14-3-3 proteins through this domain and these interactions are enhanced by polyQ expansion (Chen et al., 2003). The ataxin-1-14-3-3 interaction stabilizes phosphorylated ataxin-1 in the cytoplasm, covering its NLS, thereby blocking transport of ataxin-1 to the nucleus, disallowing NI formation and reducing toxicity (Chen et al., 2003; Lai et al., 2011).

##### Posttranslational modifications

Phosphorylation plays a critical role in mediating ataxin-1’s binding to proteins that control gene transcription and RNA splicing. AKT-mediated phosphorylation of S776 is necessary for the association of ataxin-1 with CIC and 14-3-3 proteins (Emamian et al., 2003; Lam et al., 2006; Lai et al., 2011). S776 phosphorylation and polyQ expansion allow ataxin-1 to more readily bind the spliceosome protein, RBM17 which, in turn, interferes with RNA polymerase 2 and contributes to cell death through toxic gain-of-function (Lim et al., 2008). Conversely, ataxin-1 polyQ expansion disrupts its interaction with the splicing factor, U2AF65 at phosphorylated S776 and interferes with its function (de Chiara et al., 2009). Balancing the phosphorylation of ataxin-1 by AKT, PP2A dephosphorylates S776 and regulates the interaction of ataxin-1 with RBM17 and U2AF65 (Lai et al., 2011). In short, various functions and malfunctions of ataxin-1 rely on interactions mediated by S776 phosphorylation.

Phosphorylation is not the only posttranslational modification that impacts SCA1. Unlike S776 phosphorylation, SUMOylation of expanded ataxin-1 targets it for nuclear degradation *via* the SUMO-dependent ubiquitin ligase, RNF4 (Guo et al., 2014; Wan et al., 2018). These modifications make up an additional aspect of SCA1 protein context and their variable impacts represent an interesting potential for therapeutic investigation.

To conclude, a delicate balancing act of the functions and states of modification of domains and residues surrounding the polyQ of ataxin-1 is critical for SCA1.

### Spinocerebellar ataxia type 2

#### The disease and its symptoms

While SCA2 was initially described in India in the 1960s and 1970s (Wadia and Swami, 1971), it was not until 1996 that the disease gene, *ATXN2*, along with the causative CAG expansion encoding a polyQ repeat in the causative protein, ataxin-2, were identified (Imbert et al., 1996; Pulst et al., 1996; Sanpei et al., 1996). SCA2 is characterized primarily by gait ataxia, onset coinciding with muscle cramping and other cerebellar degeneration symptoms. SCA2 signs and symptoms are almost entirely of cerebellar origin and include presentations such as appendicular ataxia, dysarthria, and ocular deficits including nystagmus and ocular dysmetria (Todi et al., 2007; Scoles and Pulst, 2018; Lieberman et al., 2019). Other frequently reported symptoms are dystonia, frontal-executive dysfunction, myoclonus, muscle spasticity, neuropathy, and slow or absent saccades (Scoles and Pulst, 2018). Like SCA1, there is major pathological involvement in the cerebellum and brainstem. There is evidence of atrophy in the pontine gray matter, middle cerebellar peduncles, cerebellar white matter and folia, and inferior olive (Todi et al., 2007; Scoles and Pulst, 2018; Lieberman et al., 2019).

**FIGURE 6:**
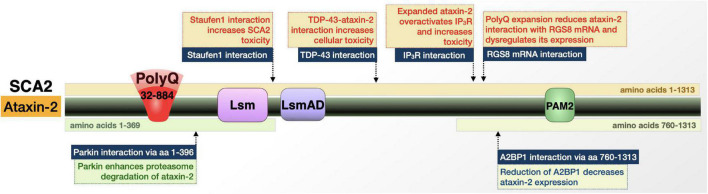
Graphic representation of the SCA2 protein, its domains, interactions, and modifications. Details are in the main text.

*ATXN2* is located on chromosome 12q24 and normally contains 15–32 CAG repeats (Todi et al., 2007; Scoles and Pulst, 2018; Lieberman et al., 2019). Pathogenicity is reached when repeats expand beyond 32 to as many as 77 repeats or more (a repeat of approximately 884 repeats was reported (Sánchez-Corona et al., 2020)) with the possibility of reduced penetrance in individuals possessing repeats of 32–34 CAG (Todi et al., 2007; Scoles and Pulst, 2018; Lieberman et al., 2019). As with other polyQ disorders, there is an inverse correlation between repeat length and age-of-onset, along with anticipation across generations. Consisting of 25 exons, *ATXN2* covers 147 megabases; the *ATXN2* transcript contains 4,699 base pairs and two in-frame start codons. The purpose of these two translational pathways remains undetermined, but translation from the first start codon produces a 144 kDa protein while translation from the second, only four codons upstream of the CAG repeat, produces an ataxin-2 protein that is 17 kDa smaller (Figure 6; Scoles and Pulst, 2018). In addition, an even smaller, 42 kDa ataxin-2 fragment was reported in postmortem patient brain extracts and in SCA2 model mice (Huynh et al., 1999, 2000; Koyano et al., 1999).

Expansion within these transcripts causes a toxic gain-of-function of ataxin-2 resulting in abnormally slow Purkinje cell firing frequency and eventual degeneration. Normally, ataxin-2 is a cytoplasmic protein that functions in RNA metabolism (Todi et al., 2007; Scoles and Pulst, 2018; Lieberman et al., 2019; Hermann et al., 1995; Huynh et al., 1999). Ataxin-2 can also localize to the trans-Golgi network (Huynh et al., 2003; Turnbull et al., 2004). These traits and others open the door for roles for ataxin-2 in stress granule formation/regulation, secretion and endocytosis, Ca^2+^ homeostasis, and apoptotic and receptor-mediated signaling. Ataxin-2 is also implicated in diseases with phenotypes that are outside of the cerebellar spectrum, most prominently L-DOPA responsive Parkinsonism and ALS (Elden et al., 2010; Scoles and Pulst, 2018).

#### Ataxin-2 protein-protein interactions dictate toxicity in SCA2 and other diseases

##### Protein-protein interactions

Ataxin-2 is implicated in several diseases and interacts with other disease proteins, including polyQ disorders like SCA1, SCA3, and HD (Ralser et al., 2005; Al-Ramahi et al., 2007; Elden et al., 2010; Scoles and Pulst, 2018). In terms of SCA2-specific context, ataxin-2 interacts with multiple RNA-binding proteins in its wild-type and polyQ-expanded forms that hint toward the role of these interactions in disease (Figure 6). Ataxin-2-binding protein 1 (A2BP1) regulates RNA splicing (Shibata et al., 2000; Scoles and Pulst, 2018). A2BP1 interacts with the C-terminal half of ataxin-2 and is present in stress granules in postmortem SCA2 patient dentate neurons and Purkinje cells. This interaction suggests that the SCA2 protein is involved in alternative splicing; postmortem patient samples point to that role occurring in a tissue-specific manner—or in a subset of RNAs—that renders specific regions/genes particularly vulnerable to ataxin-2 polyQ expansion (Shibata et al., 2000; Scoles and Pulst, 2018).

A key player in SCA2 is the interaction of ataxin-2 with Staufen1 (Stau1). Stau1 is a regulator of stress granule formation; its function in this process is increased upon ataxin-2 polyQ expansion, likely as a result of higher Stau1 levels due to expanded ataxin-2 (Paul et al., 2018; Scoles and Pulst, 2018). This information, in combination with the finding that Stau1 overexpression causes cells to constitutively generate stress granules, suggests that Stau1 interaction with polyQ-expanded ataxin-2 contributes to SCA2 (Scoles and Pulst, 2018).

Additional implications of ataxin-2-based toxicity can be found in the excitatory postsynaptic potentials. Ca^2+^ influx *via* transient receptor potential channel 3 (TRPC3) facilitates slow excitatory postsynaptic potentials. TRPC3 is gated by diacylglycerol and inositol 1,4,5- trisphosphate receptor, IP_3_R (Hartmann et al., 2011). Through an interaction with polyQ-expanded ataxin-2, IP_3_R is aberrantly activated and triggers abnormal release of Ca^2+^ from intracellular stores, disrupting neuronal signaling. This interaction and overactivation of IP_3_R by ataxin-2 appear to be specific to the polyQ-expanded protein (Liu et al., 2009).

Along with its interaction with IP_3_R, ataxin-2 is additionally involved in Ca^2+^ homeostasis and neuronal communication through its interaction with *RGS8* mRNA. *RGS8* expression is reduced in the presence of mutant ataxin-2, which may result from either mRNA degradation or *RGS8* transcript sequestration into stress granules (Liu et al., 2009; Hartmann et al., 2011; Scoles and Pulst, 2018). RGS8 is an inhibitor of metabotropic glutamate receptor type 1 (mGluR1), whose functions in Purkinje cells and motor neurons includes the regulation of local dendritic Ca^2+^ signal and slow excitatory postsynaptic potentials. The combination of IP_3_R overactivation and reduction in *RGS8* levels, resulting from interactions with polyQ-expanded ataxin-2, could impact Ca^2+^ homeostasis in Purkinje cells, disrupt neuronal signaling, and ultimately contribute to SCA2 pathology (Liu et al., 2009; Hartmann et al., 2011).

In its function as a regulator of mRNA translation, ataxin-2 assembles with polyribosomes and polyalanine (polyA)-binding protein (PABP) (Satterfield and Pallanck, 2006; Singh et al., 2021). Polyribosome assembly occurs through the ataxin-2 Lsm/Lsm-associated domain (Lsm/LsmAD), which is found in proteins associated with mRNA processing. The interaction with PABP occurs at ataxin-2’s protein interaction motif 2 (PAM2), which promotes ataxin-2’s assembly with polyribosomes. Ataxin-2’s function as a translational regulator thus occurs through binding of mRNA directly and indirectly (Satterfield and Pallanck, 2006; Singh et al., 2021). PolyQ tract expansion may interfere with these interactions and perturb their functions.

Intermediate ataxin-2 polyQ repeats modulate ALS patient survival (Chio et al., 2015; Sproviero et al., 2017). This may be at least in part through ataxin-2’s interaction with TAR DNA-binding protein of 43 kDa (TDP-43), and potentially also as a result of the functional relation of ataxin-2 with Stau1, whose protein levels are increased in ALS patients (Paul et al., 2018). Intracellular aggregates of TDP-43 are found in postmortem brains of patients with frontotemporal lobar degeneration (FTLD) and ALS (Nonhoff et al., 2007; Inagaki et al., 2021). In the case of ALS, non-pathogenic, intermediate-length ataxin-2 forms a complex with TDP-43 that causes mislocalization of ataxin-2 in a manner that is significantly associated with disease (Nonhoff et al., 2007; Inagaki et al., 2021). Down-regulating ataxin-2 could help attenuate the toxicity of TDP-43 and may make ataxin-2 a potential therapeutic target in TDP-43-related disorders, in addition to its potential benefit in SCA2 (Watanabe et al., 2020). It is becoming increasingly clear that SCA2 is rooted in disruptions in mRNA homeostasis and may also involve Ca^2+^ dysregulation.

##### Posttranslational modifications

Ataxin-2 interacts with the E3 ubiquitin ligase, Parkin, which is implicated in familial Parkinson’s disease. Parkin binds ataxin-2 directly at its N-terminal domain (Shibata et al., 2000). This interaction occurs with ataxin-2 possessing a polyQ tract in wild-type, patient, and hyper-expanded ranges, in both cell and mouse SCA2 models (Shibata et al., 2000; Huynh et al., 2007; Halbach et al., 2015). In these models, ubiquitination was more pronounced when the polyQ tract of ataxin-2 was expanded and overexpression of Parkin resulted in increased turnover of ataxin-2 (Shibata et al., 2000; Huynh et al., 2007; Halbach et al., 2015). These studies suggest that Parkin regulates ataxin-2 toxicity through ubiquitination and enhanced proteasomal degradation.

Ultimately, a mix of protein-protein interactions and posttranslational modifications impact ataxin-2 and its ability to cause malfunction and neurodegeneration in its polyQ-expanded form in SCA2 and also in other diseases of the nervous system.

### Spinocerebellar ataxia type 3

#### The disease and its symptoms

SCA3, also known as Machado-Joseph Disease, is the most common, dominantly inherited ataxia worldwide (Paulson, 2012; Ruano et al., 2014; Li et al., 2015; Nobrega et al., 2018; Matos et al., 2019). Like other polyQ SCAs, SCA3 is a progressive ataxia defined by cerebellar and brainstem dysfunction. Onset is typically in the young-to-mid adult years and initially manifests as progressive gait imbalance along with vestibular and speech difficulties. As the disease progresses, visual and oculomotor problems emerge including nystagmus, jerky ocular pursuits, slowing of saccades, disconjugate eye movements, ophthalmoplegia, and apparent bulging eyes (Lima and Coutinho, 1980; Sudarsky and Coutinho, 1995; Gwinn-Hardy et al., 2001; Paulson, 2012; Li et al., 2015; Nobrega et al., 2018; Matos et al., 2019). The most advanced stages of the disease leave the patient wheelchair bound with severe dysarthria and dysphagia (Paulson, 2012). Other symptoms can include facial atrophy, dystonia, spasticity, amyotrophy, and non-dementia mild cognitive impairment (Maruff et al., 1996; Zawacki et al., 2002; Kawai et al., 2004; Paulson, 2012). Life expectancy following disease onset is ∼20–25 years (Klockgether et al., 1998; Paulson, 2012).

**FIGURE 7:**
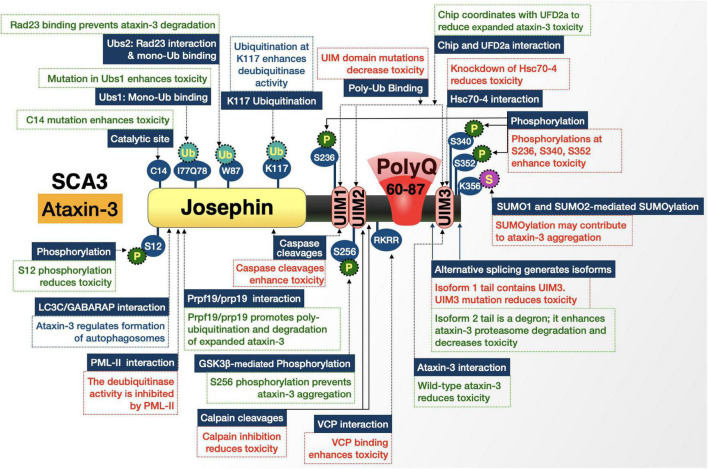
Graphic representation of the SCA3 protein, its domains, interactions, and modifications. Details are in the main text.

CAG expansion occurs in a gene first identified in 1994 as *MJD1*, now referred to as *ATXN3*, that encodes the deubiquitinating enzyme (DUB) ataxin-3 (Figure 7; Kawaguchi et al., 1994). Ataxin-3 accumulates within NI of specific populations of neurons in several brain regions that experience degeneration, particularly pontine neurons, dentate nuclei, thalamus, substantia nigra, globus pallidus, cranial motor nerved nuclei, and the striatum (Paulson et al., 1997; Schmidt et al., 1998; Rub et al., 2006; Paulson, 2012; Li et al., 2015; Nobrega et al., 2018; Matos et al., 2019). The increase in CAG tract length in *ATXN3* causes an expanded polyQ in ataxin-3 that misfolds, aggregates, and precipitates NI formation in affected regions (Kawaguchi et al., 1994; Takiyama et al., 1995; Paulson, 2012; Li et al., 2015; Nobrega et al., 2018; Matos et al., 2019). Whereas normal alleles possess 12–43 repeats, pathogenic *ATXN3* alleles contain 60–87 repeats with milder forms of ataxia and restless leg syndrome appearing in patients with mid-range repeats (Ranum et al., 1995; Paulson, 2012; Li et al., 2015; Nobrega et al., 2018; Matos et al., 2019). In the fully penetrant range, larger repeats (often > 73) are associated with a worse degree of pyramidal signs and dystonia (Jardim et al., 2001) while repeats on the lower end of the pathogenic range are associated with peripheral neuropathy (Durr et al., 1996; Schöls et al., 1996; Paulson, 2012).

Ataxin-3 is a widely expressed 42 kDa protein. Like other disease proteins covered here, mutant ataxin-3 leads to selective tissue vulnerability that cannot be explained by protein expression (Paulson, 2012; Li et al., 2015; Nobrega et al., 2018; Matos et al., 2019). As a DUB, ataxin-3 plays a role in the clearance of misfolded proteins by the ubiquitin-proteasome system (UPS); it shows neuroprotective effects against other polyQ proteins, as well as itself, in model systems; and has also been implicated in DNA damage repair (Warrick et al., 2005; Paulson, 2012; Tsou et al., 2013, 2016; Li et al., 2015; Sutton et al., 2017; Nobrega et al., 2018; Matos et al., 2019). While the exact roles of ataxin-3 remain unclear, it is thought that the expansion of its polyQ domain burdens the protein quality control of cells in affected regions (Paulson, 2012; Li et al., 2015; Nobrega et al., 2018; Matos et al., 2019). Ataxin-3 consists of several domains and splice isoforms that are important to its native function and SCA3 pathogenesis, discussed next.

#### The pathogenicity of ataxin-3 is regulated by the modification of its domains and their partners

##### Posttranslational modifications and proteolytic processing

Various studies have explored ataxin-3’s roles in cellular pathways and processes (Warrick et al., 2005; Winborn et al., 2008; Costa and Paulson, 2012; Tsou et al., 2013, 2015b; Matos et al., 2019; Weishaupl et al., 2019; Dantuma and Herzog, 2020). Still a clear function besides it being a DUB remains elusive and likely reflects pleiotropic activities by this enzyme that, at least in mice, is not essential (Schmitt et al., 2007; Switonski et al., 2011). Posttranslational modifications are an important part of protein context in SCA3; the first covered here is phosphorylation, followed by SUMOylation and ubiquitination.

The pathogenic effects of expanded ataxin-3 are influenced by phosphorylation at S12 in several models. In cortical neurons, S12 phosphorylation reduced pathogenic ataxin-3-dependent synapse loss and toxic aggregation. This protection was mimicked in a lentiviral-based rat model of SCA3. The ameliorative effects of S12 phosphorylation could be explained by the fact that constitutive phosphorylation at this site reduces DUB activity and could perhaps limit pathogenic ataxin-3 toxic gain-of-function (Matos et al., 2016). S256 on ataxin-3 is another site of phosphorylation that appears to limit its toxicity. S256 is phosphorylated by glycogen synthase kinase 3β (GSK3β) and prevents ataxin-3 aggregation; mutations at this site to disable phosphorylation cause increased ataxin-3 aggregation (Fei et al., 2007). However, the effects of ataxin-3 phosphorylation are not exclusively beneficial. Phosphorylation at residues S236, S340, and S352 enhance ataxin-3 nuclear localization and repress its functions as a transcriptional regulator (Tao et al., 2008; Mueller et al., 2009). [Nuclear presence of pathogenic ataxin-3 is especially toxic (Schmidt et al., 1998; Bichelmeier et al., 2007; Costa and Paulson, 2012; Ristic et al., 2018; Matos et al., 2019)].

Ataxin-3 is SUMOylated by SUMO1 and SUMO2 at residue K356 (Zhou et al., 2013; Almeida et al., 2015). SUMOylation increases ataxin-3’s affinity for VCP, a hexameric triple ATPase that is suspected to seed pathogenic ataxin-3 aggregation and could also negatively impact VCP’s function in mediating ER-associated degradation (ERAD) (Wang et al., 2006; Zhong and Pittman, 2006; Almeida et al., 2015).

Ubiquitination additionally influences polyQ-expanded ataxin-3 toxicity. C-terminus of Hsc70-interacting protein (CHIP), in coordination with the ubiquitin chain assembly factor (E4), known as E4B/UFD2a, interacts with ataxin-3 to promote its ubiquitination and degradation (Matsumoto et al., 2004; Jana et al., 2005; Miller et al., 2005). In an expanded polyQ state, ubiquitination may be protective against ataxin-3-induced degeneration, as evidenced by studies utilizing *Drosophila* models of SCA3 (Matsumoto et al., 2004; Tsou et al., 2013). One study showed that E4B expression led to the suppression of neurodegeneration (Matsumoto et al., 2004). Other studies, looking at ubiquitination on K117, found that this modification protected against degeneration induced by toxic polyQ species in flies (Tsou et al., 2013, 2015b; Sutton et al., 2017). In these latter studies ubiquitination, including on K117, did not increase the degradation of ataxin-3; instead, the proposed mechanism was one whereby ubiquitinated ataxin-3 is more active as an enzyme and able to better protect against polyQ toxicity by enhancing the production of heat shock proteins (Tsou et al., 2013, 2015b; Sutton et al., 2017). The precise mechanisms underscoring these effects are unclear.

Lastly, ataxin-3 is a target of proteolytic processing from caspases and calpains (Costa and Paulson, 2012). Caspase cleavages lead to the separation of the polyQ tract of ataxin-3 from the rest of the protein and enhance its aggregation in cultured cells and toxicity in *Drosophila* (Berke et al., 2004; Jung et al., 2009; Costa and Paulson, 2012). Recently, calpain cleavage was additionally shown to enhance pathogenesis in SCA3 model mice (Hubener et al., 2013; Harmuth et al., 2018).

##### Protein-protein interactions

Several regions and domains comprise ataxin-3 and contribute to its protein context. We begin at the N-terminal catalytic (Josephin) domain, which houses the catalytic site that is necessary for the DUB activity and ubiquitin-binding sites (UbS) 1 and 2. An intact catalytic site is necessary for ataxin-3’s neuroprotective functions against polyQ proteins and its loss through mutation of C14 enhances toxicity in *Drosophila* models of SCA3 (Warrick et al., 2005; Tsou et al., 2013, 2015b; Sutton et al., 2017; Johnson et al., 2020).

Ubs2 facilitates not only ataxin-3’s interaction with ubiquitin to aid cleavage, but is also the site where the proteasome-associated protein, Rad23 binds ataxin-3 (Blount et al., 2014; Tsou et al., 2015b; Sutton et al., 2017). The binding of Rad23 is necessary for ataxin-3’s ability to upregulate the expression of the co-chaperone protein DnaJ-1, which functions as a self-regulating, protective pathway—DnaJ-1 upregulation suppresses polyQ toxicity (Tsou et al., 2013, 2015b; Sutton et al., 2017). The ataxin-3-Rad23 interaction also stabilizes the SCA3 protein by decelerating its proteasomal turnover; this process leads to higher levels of pathogenic ataxin-3 in *Drosophila*, but the higher levels are seemingly negated by the auto-protective role of ataxin-3 (Blount et al., 2014; Sutton et al., 2017). (Our ongoing, unpublished work indicates that Ubs1 also plays a protective role, potentially through mechanisms that involve pathogenic ataxin-3 binding certain ubiquitin species but not being able to process them).

Moving towards the C-terminus, we find three ubiquitin-interacting motifs (UIMs) that surround the polyQ tract; they bind poly-ubiquitin and assist ataxin-3 in its preference for cleaving K63-linked chains (Winborn et al., 2008). When mutations are introduced to the UIMs that prevent their binding to poly-ubiquitin, they reduce expanded ataxin-3 aggregation and toxicity in *Drosophila* models of SCA3 implying that, upon expansion, the UIMs enhance ataxin-3-related pathogenesis. This latter study suggested that UIM-mediated toxicity occurs through two mechanisms: the first is enhancement of the ability of ataxin-3 proteins to bind one another, increasing their aggregation and toxicity (Johnson et al., 2020); the second is the interaction of ataxin-3 with the *Drosophila* heat shock cognate 70-4 (HSC70-4), ortholog to human heat shock protein A8 (HSPA8), through the UIMs. HSC70-4 appears to enhance ataxin-3 aggregation and pathogenicity, although this effect may be indirect (Johnson et al., 2020).

The next region of ataxin-3 that reportedly plays a role in toxicity is the one that mediates the direct binding of ataxin-3 with VCP *via* the VCP-binding domain, or VBM. Like the UIMs and HSC70-4 interaction, the VBM on ataxin-3 and its interaction with VCP enhance the toxicity of polyQ-expanded ataxin-3 in *Drosophila*. This enhancement may occur through VCP’s ability to form homohexamers that bind multiple ataxin-3 proteins and bring them into closer proximity, seeding aggregation (Ristic et al., 2018; Johnson et al., 2021). This notion was validated in studies where displacement of expanded ataxin-3 from endogenous VCP using a decoy protein improved disease phenotypes in a dose-dependent manner in *Drosophila* (Johnson et al., 2021).

Lasty, different isoforms of ataxin-3 arise from alternative splicing (Kawaguchi et al., 1994; Goto et al., 1997; Harris et al., 2010). While the isoform covered here (with three UIMs) is the predominant form, there is another version of ataxin-3 with an alternative C-terminus, lacking UIM3. (This isoform was the originally cloned version of *ATXN3*.) The alternative isoform is no less toxic than the predominant version in fly models of SCA3, but it is present at markedly lower protein levels because of enhanced proteasomal degradation due to its C-terminus serving as a degron sequence (Johnson et al., 2019; Blount et al., 2020).

In sum, the toxicity that emanates from the expanded polyQ tract of ataxin-3 is tightly controlled by neighboring and distant regions that represent potential therapeutic entry points for SCA3.

### Spinocerebellar ataxia type 6

#### The disease and its symptoms

**FIGURE 8:**
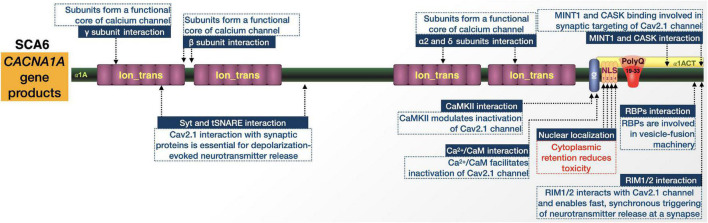
Graphic representation of the SCA6 proteins, domains, and interactions. Details are in the main text.

SCA6 is caused by relatively short CAG expansions in the bicistronic *CACNA1A* gene that encodes the α1A subunit of the P/Q-type voltage-gated Ca^2+^ channel (Cav2.1) and the transcription factor α1ACT (Figure 8; Todi et al., 2007; Du and Gomez, 2018; Lieberman et al., 2019). Identified in 1997, it is the first and thus far only polyQ disease attributed to mutations in an ion channel-encoding gene (Zhuchenko et al., 1997).

The disease has variable frequency in various populations worldwide and is particularly prominent in the Netherlands and Japan. Clinically, SCA6 is a pure cerebellar ataxia with typical appearance later in life. Patients show pure cerebellar dysfunction, slowly progressive gait ataxia and imbalance, dysarthria, and late-onset nystagmus. While the age of onset can range anywhere from 19 to 71 years, the mean age-of-onset is 43 to 52 and is inversely correlated with the size of the expansion. After onset, SCA6 manifests more slowly than other SCAs and does not usually shorten the patient’s lifespan, but even with slow progression there can be large variances in symptom severity (Todi et al., 2007; Ruano et al., 2014; Du and Gomez, 2018; Lieberman et al., 2019). In addition to SCA6, mutations in *CACNA1A* are associated with at least two dominantly inherited disorders, episodic ataxia type 2 and familial hemiplegic migraine, both of which clinically overlap with SCA6 (Ophoff et al., 1996; Todi et al., 2007; Du and Gomez, 2018).

The pathological presentation of SCA6 is defined by loss of Purkinje cells primarily in the cerebellar vermis with limited degeneration in associated cortical and cerebello-olivary regions (Todi et al., 2007; Du and Gomez, 2018; Lieberman et al., 2019). The remaining Purkinje cells in the most degenerated regions undergo extensive morphological changes, including heterotypic nuclei, unclear cytoplasmic outline, somatic sprouts, dendritic swelling with increased numbers of spine-like protrusions, and disorganization of axonal arrangements (Du and Gomez, 2018).

*CACNA1A* is on chromosome 19p13 and contains 47 exons, the last of which contains the CAG triplet repeat. Normally this repeat is 4–18 CAG long but is expanded to 19–33 in SCA6 (Todi et al., 2007; Du and Gomez, 2018; Lieberman et al., 2019). SCA6 shows variable penetrance within its pathological range, where heterozygous individuals with 19 repeats are usually asymptomatic while those possessing 20 or more repeats show full penetrance (Du and Gomez, 2018).

*CACNA1A* has an internal ribosomal entry site (IRES) that allows it to encode two different proteins. They have distinct functions and contain overlapping open-reading frames within the same mRNA. The first is the α1A subunit of the Cav2.1 channel. The second is the transcription factor, α1ACT. Both proteins are expressed abundantly in Purkinje cells and α1ACT with an expanded polyQ is toxic in tissue culture in a manner that is dependent on nuclear localization. α1ACT coordinates the expression of genes involved in cerebellar development. In addition to the expanded polyQ and its NLS, it contains regions typically associated with channel inactivation and modulation by intracellular signaling (Todi et al., 2007; Du and Gomez, 2018; Lieberman et al., 2019). One hypothesis is that SCA6 is an ion channel disorder and the polyQ expansion causes ataxia by altering Ca^2+^ channel function; investigations into this theory have yielded conflicting findings (Du and Gomez, 2018). Other evidence predicates the nuclear accumulation of α1A subunits, which suggests that pathogenesis could relate to the nuclear accumulation of expanded polyQ proteins/fragments that lead to transcriptional dysregulation, as observed in other polyQ disorders (Kordasiewicz et al., 2006). Accumulation of polyQ-expanded fragments may additionally promote a toxic gain-of-function that exacerbates disease progression through the sequestration of housekeeping genes and transcription factors, disruption of UPS, DNA damage, or induction of apoptosis (Lipinski and Yuan, 2004; Ross et al., 2004). The more recent findings with the transcription factor, α1ACT, also point to a mechanism of toxicity primarily rooted in transcriptional dysregulation (Ophoff et al., 1996; Du and Gomez, 2018).

#### Proteins implicated in SCA6

##### Protein-protein interactions and sub-cellular localization

Information on protein context in SCA6 from the perspective of α1ACT is limited by comparison to other polyQ diseases (Figure 8). Upon polyQ expansion, α1ACT loses its transcription factor function and neurite outgrowth properties leading to cell death in cell culture, and ataxia and cerebellar atrophy in transgenic mice (Du et al., 2013). There is also evidence that SCA6 toxicity is dependent on the nuclear localization of pathogenic α1ACT, mediated by NLS (Kordasiewicz et al., 2006; Ishiguro et al., 2010).

Few proteins are reported to alter α1ACT toxicity. One is the J-protein co-chaperone DnaJ-1. Expression of DnaJ-1 reduced polyQ-expanded α1ACT degeneration and lethality in *Drosophila* models of SCA6, along with a reduction in aggregation of the toxic protein (Tsou et al., 2015a). The same goes for the nuclear importer karyopherin α3. Mutating this protein in *Drosophila* also reduced pathogenic α1ACT toxicity (Tsou et al., 2015a). Clearly, there is much room to expand the understanding of α1ACT’s protein context.

While little is known about the interactions of α1ACT and their impact on SCA6, *CACNA1A* pathogenic variants have been associated with various neurological disorders including Developmental and Epileptic Encephalopathy (DEE), episodic ataxia type 2 (EA2), familial hemiplegic migraine type 1 (FHM1), and SCA6 (Pietrobon, 2010). PolyQ expansions in any of the Cav2.1 proteins encoded by these pathogenic variants may change the conformation of the channel and affect its protein interactions and channel function. Further studies into known Cav2.1 protein-protein interactions could prove useful for understanding SCA6, if placed in the context of an expanded polyQ. These interactions include Syt and tSNARE (Cohen-Kutner et al., 2010; Rajakulendran et al., 2012), Ca^2+^/calmodulin-dependent protein kinase II (CaMKII) (Jiang et al., 2008; Magupalli et al., 2013), Ca^2+^/CaM-IQ domain interactors (Lee et al., 2003; Kim et al., 2008), RIM1/2 (Kaeser et al., 2011), MINT1 (Maximov et al., 1999), Rim-Binding Proteins (RBPs) (Hibino et al., 2002), CASK proteins (Maximov et al., 1999), and internal interactions between the α1 and auxiliary α2/δ and β subunits (Opatowsky et al., 2004; McKeown et al., 2006).

Elucidating the differences in interactions between α1A and α1ACT, including between their wild-type and polyQ-expanded forms, will help in determining what contributes to SCA6-specific pathology.

### Spinocerebellar ataxia type 7

#### The disease and its symptoms

**FIGURE 9:**
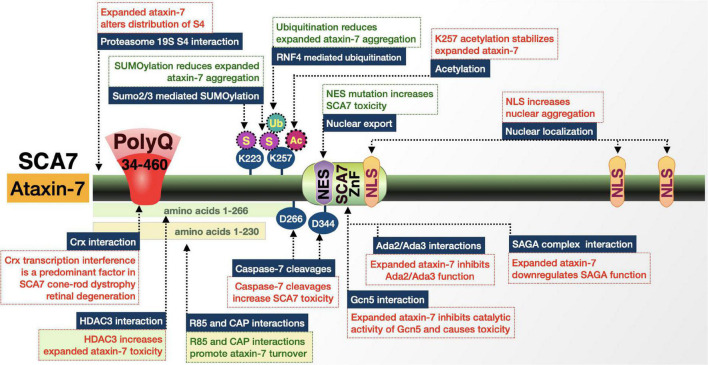
Graphic representation of the SCA7 protein, its domains, interactions, and modifications. Details are in the main text.

SCA7 is caused by a polymorphic triplet-repeat expansion in the *ATXN7* gene encoding ataxin-7 (ATXN7) located on chromosome 3p14.1 (Figure 9; David et al., 1997; Michalík et al., 1999; Niewiadomska-Cimicka and Trottier, 2019). The gene and expansion were identified in 1997, making SCA7 the eighth of nine known polyQ disorders (David et al., 1997). Clinical presentation is similar to SCAs 1, 2, and 3: spastic ataxia, dysarthria, dysphagia, slow eye movement, opthalmoplegia, prominent hyperflexia with crossed supraclavicular, pectoral, and hip adductor reflexes, spasticity, and pyramidal signs. What makes SCA7 unique is that it is the only polyQ disorder that is additionally characterized by retinal degeneration. Visual impairment begins with cone photoreceptor degeneration that progresses toward cone-rod dystrophy and eventually complete blindness. Postmortem pathology from patient cerebella shows substantial loss in the Purkinje cell layer, dentate nuclei, and to a lesser extent the granule cell layer. The initial, ataxia-like symptoms that appear early in SCA7 match well with the primary neuropathology, then become increasingly diverse; the degenerative process becomes more widespread as the disease progresses (Niewiadomska-Cimicka and Trottier, 2019).

Repeat expansions in *ATXN7* have some of the widest ranges among polyQ disorders. *ATXN7* is widely expressed in neuronal and non-neuronal tissue. The triplet-repeat tract of wild-type *ATXN7* typically has 4–35 repeats, with an estimated 70–80% of all *ATXN7* genes carrying 10 triplets (David et al., 1997; Niewiadomska-Cimicka and Trottier, 2019). Alleles with 28–35 repeats are prone to expansion and repeats of 34–36 are associated with reduced penetrance as well as mild- and late-onset disease. SCA7 repeats show the highest tendency to expand upon transmission and show strong anticipation—while 36 repeats are the pathological threshold, expansions can reach a length of 460. This means that there is a wide range of fully penetrant manifestations of SCA7: 36–55 repeats present as adult-onset SCA7; > 70 repeats present as a juvenile-onset form of SCA7 with accelerated progression; and > 100 repeats present as a severe infantile disease with death in the first few months or years of life (David et al., 1997; Niewiadomska-Cimicka and Trottier, 2019). Longer repeats are not only inversely correlated with the age-of-onset, but also result in an earlier appearance of retinal degeneration and vision loss prior to cerebellar ataxia (Niewiadomska-Cimicka and Trottier, 2019).

ATXN7 is a member of the multiprotein Spt-Ada-Gcn5 acetyltransferase (SAGA) complex (Burke et al., 2013; Cloud et al., 2019; Niewiadomska-Cimicka and Trottier, 2019). SAGA is responsible for interactions with transcriptional machinery and chromatin modifications through both histone deacetyltransferase (HAT) and DUB activities. Within this complex, ATXN7 has several roles. The first is in the DUB module where it anchors the DUB (USP22) to the rest of SAGA. The second is as a functional scaffold of SAGA that regulates H2B ubiquitination and is important for Purkinje cell differentiation and the maintenance of differentiated retinal photoreceptors (Niewiadomska-Cimicka and Trottier, 2019).

ATXN7 is localized to the cytoplasm and nucleus of neurons and the ratio between the two compartments varies by brain region. It is also present in all retinal neurons. PolyQ-expanded ATXN7 forms large NI and aggregates across degenerating and spared brain regions, but does seem to accumulate faster in vulnerable nuclei, indicating that accumulation, not localization, is a better indicator of toxicity. The polyQ domain is on the N-terminal portion and is followed by a small proline repeat that resembles the polyQ/proline-rich regions of HD and other transcription factors (David et al., 1997; Niewiadomska-Cimicka and Trottier, 2019). Additional domains include three NLS and one NES. The pathogenic effects of polyQ-expanded ATXN7 in mice are both cell autonomous and non-cell autonomous (Young et al., 2007; Niewiadomska-Cimicka and Trottier, 2019).

#### Ataxin-7 domains and interactions give clues toward SCA7 pathogenicity and therapeutics

##### Proteolytic processing and sub-cellular localization

While transcriptional dysregulation is a factor in SCA7 because of the residency of ATXN7 in SAGA, there are also domains and protein-protein interactions involving ataxin-7 that influence disease severity (Figure 9). Once such example is the cleavage of expanded ataxin-7 by caspase-7. Aspartic acid residues at positions 266 and 344 of ataxin-7 are caspase-7 cleavage sites (Young et al., 2007; Guyenet et al., 2015). Caspase-7 cleavage appears critical in SCA7. On the one hand, preventing processing through mutation of polyQ-expanded ataxin-7 mitigated cell death, aggregate formation, and transcriptional interference that is observed in SCA7; prevention of cleavage at D266 also reduced neurodegeneration, extended lifespan, and improved motor performance in SCA7 model mice (Young et al., 2007; Guyenet et al., 2015). On the other hand, expression of the caspase-7 truncation product of ataxin-7 with two different pathogenic repeat lengths, which also removes ataxin-7 NES and NLS, increased cellular toxicity. One hypothesis is that caspase-7 cleaves ataxin-7 in the nucleus rather than the cytoplasm, separating the polyQ tract from the NES and thus preventing the nuclear export of ataxin-7 (Young et al., 2007; Guyenet et al., 2015). Nuclear retention could lead to the accumulation of toxic fragments that impair ataxin-7’s normal interactions with SAGA.

##### Posttranslational modifications

Accumulation of expanded ataxin-7 fragments that result from cleavage events is associated with increased levels of acetylation at K257. This residue, which is also the site of additional modifications, is close to one of the two caspase-7 cleavages sites. Acetylation of K257 is associated with reduced degradation of ataxin-7 by macroautophagy, enhancing its toxicity (Mookerjee et al., 2009).

In addition to acetylation, K257 of ataxin-7 is SUMOylated and poly-SUMOylated. SUMOylation has a contrasting effect compared to acetylation. SUMO2 is added to polyQ-expanded ataxin-7 and leads to the recruitment of SUMO-targeted ubiquitin ligase, RNF4. Ataxin-7 is ubiquitinated by RNF4 and, following the recruitment of clastosomes, is degraded by the proteasome. SUMO2 colocalizes with ataxin-7 NIs and while SUMOylation at K257 seems to promote the clearance of expanded ataxin-7, it is possible that chronic expression of this polyQ protein could produce sufficient misfolded species to eventually overwhelm the SUMO pathway and clastosomes, especially as they become compromised with age (Janer et al., 2010; Marinello et al., 2019). An overwhelmed clearing system may help explain the delayed onset of this disease.

##### Protein-protein interactions

The interface between ataxin-7 and SAGA presents various interactions that play a role in SCA7. Upon polyQ expansion, ataxin-7′s interaction with SAGA alters its function through the sequestration of SAGA components into NIs (Yvert et al., 2000, 2001; Yoo et al., 2003; Lan et al., 2015; Yang et al., 2015), compromising SAGA integrity (McMahon et al., 2005; Palhan et al., 2005), and aberrantly interacting with SAGA components and associated transcription factors (Burke et al., 2013). For example, the cone-rod homeobox protein (Crx) is a transcription factor that interacts with SAGA through ataxin-7 (Lines et al., 2002; Chen et al., 2004). The Crx-SAGA interaction controls Crx-dependent gene activation in the retina (Helmlinger et al., 2004; Palhan et al., 2005). PolyQ expansion of ataxin-7 disrupts this pathway leading to the downregulation of retinal-specific genes and contributing to disease progression, as documented by transgenic SCA7 knock-in mice (La Spada et al., 2001; Lines et al., 2002; Chen et al., 2004; Helmlinger et al., 2004; Palhan et al., 2005). These and other findings (Burke et al., 2013) suggest that polyQ-expanded ataxin-7 dominantly inhibits the function of the SAGA complex and help to explain the prominence of transcriptional dysregulation in SCA7.

There are pertinent protein-protein interactions of ataxin-7 outside of SAGA that play additional roles in SCA7. One is histone deacetylase 3 (HDAC3), whose activity increases in SCA7 mouse retinas and whose interaction with ataxin-7 stabilizes the protein in a deacetylase-independent manner (Duncan et al., 2013). Another interaction is with the Sorbin and SH3 Domain-Containing protein 1 (*SORBS1/SH3P12*) gene products, R85 and Cbl-associated protein (CAP), in both a wild-type and polyQ-expanded state (Lebre et al., 2001). It is suggested that in a wild-type state, this interaction helps to facilitate the physiological regulation, ubiquitination, and degradation of ataxin-7; however, when ataxin-7 is expanded, ubiquitinated proteins accumulate in NIs in SCA7 brains, suggesting that the normal function of these gene products is disrupted and that they may contribute to SCA7 (Lebre et al., 2001).

The final ataxin-7 interaction that we cover here is with the ATPase subunit S4 of the 19S regulatory complex of the proteasome. Uncovered through a yeast two-hybrid assay, the ataxin-7-S4 interaction is inversely correlated with the length of the polyQ tract. S4 may increase the ubiquitin-mediated proteasomal degradation of ataxin-7 and other substrates. As the polyQ tract of ataxin-7 expands and its interaction with S4 weakens, it may create abnormalities in the recognition or degradation of ubiquitin protein conjugates by the 26S proteasome and contribute to accumulation of ataxin-7/ubiquitin-protein conjugates in SCA7 (Matilla et al., 2001).

As with the other polyQ family disorders, insights into SCA7 pathogenesis and potential therapeutics will continue to emerge from a detailed understanding of the pleiotropic interactions and modifications that are linked to its polyQ repeat.

### Spinocerebellar ataxia type 17

#### The disease and its symptoms

**FIGURE 10:**
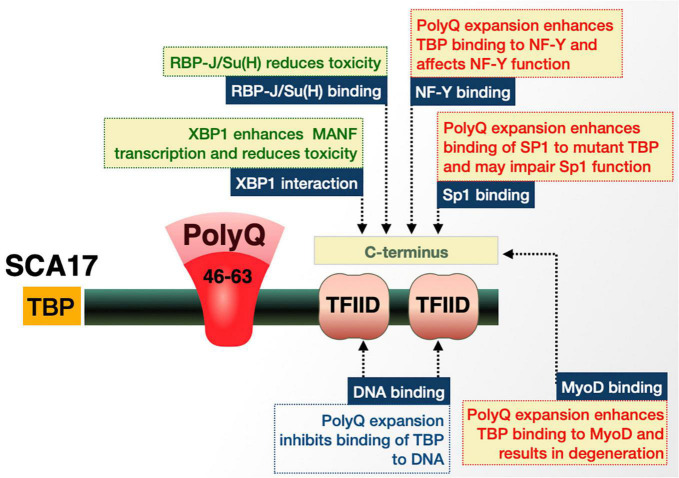
Graphic representation of the SCA17 protein, its domains, and interactions. Details are in the main text.

The most recently discovered polyQ disorder, SCA17 is a rare disease accounting for approximately 0.5-1% of dominantly inherited ataxias. It is caused by a polyQ expansion in the TATA-box binding protein (TBP), an essential transcription initiation factor (Figure 10; Koide et al., 1999; Nakamura et al., 2001; Zühlke and Bürk, 2007). Clinically, SCA17 can resemble the presentation of Parkinson’s disease and HD, and includes symptoms ranging from psychosis to multitudinous cerebellar syndromes. Patients experience many of the early signs of ataxia with gait imbalance, dysarthria, dystonia, and choreic movements, but also encounter intellectual decline, dementia, psychiatric abnormalities, seizures, and basal ganglia symptoms that can help distinguish it from other SCAs and HD. At the pathological level, SCA17 manifests as marked cerebellar atrophy and degeneration, as well as moderate and diffuse cortical, subcortical, and brainstem atrophy. Purkinje cell loss and gliosis are also prominent (Zühlke and Bürk, 2007; Toyoshima and Takahashi, 2018; Liu et al., 2019).

The 18,528-bp *TBP* gene is located on chromosome 6q. The 1,867-bp transcript spanning exons 2–8 encodes TBP (Zühlke and Bürk, 2007). The polyQ repeat is encoded by exon 3; wild-type alleles typically have 25–42 repeats, with the most common range in humans being 32–39 (Zühlke and Bürk, 2007; Toyoshima and Takahashi, 2018; Liu et al., 2019). The TBP polyQ contains two polymorphic (CAG)n stretches interrupted by polyQ-encoding CAAs in the following pattern: (CAG)3 (CAA)3 (CAG)n CAA CAG CAA (CAG)n CAA CAG. These interruptions stabilize the repeat sequence and it is thought that intergenerational instability and anticipation result from the loss of the interrupting CAA CAG CAA element and fusion of the (CAG)n blocks. Stabilization by CAA interruptions—in tandem with the narrow gap between normal and abnormal repeats—makes it difficult to determine an exact pathological cutoff. The current understanding is that the range for SCA17 is 46–55 repeats, with 43–48 repeats constituting an intermediate range with incomplete penetrance (Zühlke and Bürk, 2007; Toyoshima and Takahashi, 2018; Liu et al., 2019). Juvenile-onset SCA17 has been reported in patients with repeats exceeding 62 who experience ataxia, intellectual deterioration, muscle weakness, growth retardation, faster disease progression, and early death (Liu et al., 2019).

TBP is a saddle-shaped protein that is recruited to the basal promoter elements of RNA polymerases I, II, and III and can directly bind the TATA boxes on DNA or, in the case of TATA-less promoters, has protein-protein interactions to initiate transcription. TBP is implicated in neurodegenerative disorders beyond SCA17 including HD, SCA1, SCA2, and SCA3 (Zühlke and Bürk, 2007; Roshan et al., 2017; Liu et al., 2019). TBP functions as the “nucleus” of the initiation complex and provides docking sites for several additional transcription factors (Zühlke and Bürk, 2007; Liu et al., 2019).

TBP has a variable N-terminal domain and a highly conserved C-terminal domain. The C-terminal portion houses the DNA-binding domain, while the N-terminal portion assists in modulating DNA-binding activity. The polyQ stretch is on the N-terminus. As the offending repeat resides on the N-terminal half of the protein, it has been proposed that sub-regions surrounding the polyQ synergistically promote the formation of the TBP-DNA complex and that neurodegeneration upon polyQ expansion arises from a toxic gain-of-function. Multiple models show age-dependent accumulation of polyQ-expanded TBP aggregates in neuronal nuclei and cerebellar degeneration, especially Purkinje cell death, but the role of aggregates in degeneration is unclear (Zühlke and Bürk, 2007; Liu et al., 2019).

#### TBP and its role in SCA17 and other neurodegenerative diseases

##### DNA- and protein-protein interactions

Dysfunctions in TBP (Figure 10) result in broad clinical presentations ranging from Parkinson’s to Huntington’s to psychiatric symptoms, impacting pathologies beyond SCA17 (Zühlke and Bürk, 2007; Toyoshima and Takahashi, 2018; Liu et al., 2019). In *Drosophila*, loss of TBP expression not only caused age-related neurodegeneration, but also exacerbated polyQ expansion-induced retinal degeneration in models of SCA3 and HD (Hsu et al., 2014). Findings like these suggest that TBP plays a larger role in polyQ-induced degeneration.

One element of TBP protein context that is altered and important with polyQ expansion is its structural conformation. A conformational change that occurs upon polyQ expansion may disrupt one of TBP’s native functions, DNA-binding. PolyQ expansion reduces the binding of TBP to DNA *in vitro*. Also, TBP fragments harboring an expanded polyQ tract, but lacking an intact C-terminal DNA-binding domain, were identified in transgenic SCA17 mice. In addition to reduced DNA binding, polyQ-expanded TBP with a deletion in the DNA-binding domain formed nuclear aggregates and inhibited TATA-dependent transcription in cultured cells and caused early death and NI in transgenic mice (Friedman et al., 2008). These findings suggest that the expanded polyQ tract of TBP negatively affects its ability to bind DNA and that this disruption can induce SCA17.

Besides changes in DNA-binding, TBP also has several new or altered protein-protein interactions that occur because of polyQ expansion. One example is the transcription factor, nuclear factor-Y (NF-Y) (Friedman et al., 2008). In transgenic mice, polyQ-expanded TBP bound more tightly to NF-Y and sequestered it into NI, possibly altering its function as a master regulator of the chaperone system and impairing NF-Y-mediated expression of chaperones like Hsp70, Hsp25, and HspA5 (Huang et al., 2011). PolyQ-expanded TBP also abnormally binds to another transcription factor, Sp1, and sequesters it away from its normal function in a manner similar to NF-Y. In SCA17 knock-in mice, the mutant TBP-Sp1 interaction led to reduced transcription of *Inpp5a*, a gene that is critical for Purkinje neuron maintenance and neurite growth, and whose knockout in wild-type mice results in Purkinje neurodegeneration (Shah et al., 2009; Liu et al., 2020). A final example of the polyQ expansion altering TBP’s interaction with proteins is yet another transcription factor, RBP-J/Su(H) (Recombination signal-binding protein for immunoglobulin kappa J region). This transcription factor participates in Notch signaling and was shown in *Drosophila* to interact more efficiently with polyQ-expanded TBP compared to wild-type TBP. Its dysfunction and subsequent alteration of the Notch signaling pathway contributed to polyQ-expanded TBP-induced phenotypes in this model (Ren et al., 2011). These examples indicate a trend of enhanced complexing of polyQ-expanded TBP with various factors which, like NF-Y, Sp1, and RBP-J/Su(H), could be sequestered away from normal functions and contribute to SCA17.

While polyQ expansion increases several TBP interactions, there are also cases where it has the opposite effect. For MANF (Mesencephalic astrocyte-derived neurotrophic factor), an ER stress inducible protein that is enriched in Purkinje cells, the connection to TBP is through a shared interaction with the transcription factor, XBP1 (X-Box-binding protein 1). XBP1 recognizes the ER stress response element in the promoter of *MANF*. XBP1 is also normally present in the same transcriptional complex as TBP to help mediate the expression of *MANF*; however, polyQ-expanded TBP has decreased association with XBP1, leading to reduced expression of *MANF* and a reduced ER stress response (Hetz, 2012; Yang et al., 2014). Another normal interaction that is impacted by polyQ expansion in TBP is with the muscle-specific transcription factor MyoD (Myogenic differentiation 1). The TBP-MyoD interaction stabilizes MyoD’s binding to DNA; however, this partnership is reduced upon TBP polyQ expansion, causing decreased transcriptional activity, which negatively impacted muscle-specific gene expression and led to muscle degeneration in SCA17 knock-in mice (Huang et al., 2015).

Continued investigations into the protein context of SCA17 are bound to gather revelatory information that can help the field understand its pathogenesis and progression and, due to the apparent involvement of TBP in other disorders, may also prove informational more widely.

## The intersections of polyglutamine diseases

The above studies identified potential mechanistic intersections among some polyQ diseases; their continued investigation will strengthen our general understanding of this family of incurable disorders. The next few paragraphs focus on some such intersections.

### Posttranslational modifications

Posttranslational modifications are common elements of protein context among the polyQ diseases. SUMOylation, phosphorylation, and acetylation all play roles in the toxicity of multiple disease proteins; but, the outcomes from these modifications are not always the same. For example, SUMOylation reduces polyQ toxicity in HD, SCA1, and SCA7 (Steffan et al., 2004; Janer et al., 2010; Guo et al., 2014; Wan et al., 2018; Marinello et al., 2019); however, SUMOylation of ataxin-3 increases its affinity to VCP, a protein that enhances ataxin-3 aggregation and exacerbates toxicity (Wang et al., 2006; Zhong and Pittman, 2006; Zhou et al., 2013; Almeida et al., 2015; Ristic et al., 2018; Johnson et al., 2021). Similarly, AKT-mediated phosphorylation reduces toxicity in models of HD and SBMA (Lin et al., 2001; Humbert et al., 2002; LaFevre-Bernt and Ellerby, 2003; Gauthier et al., 2004; Luo et al., 2005; Pardo et al., 2006; Schilling et al., 2006; Metzler et al., 2007, 2010; Colin et al., 2008; Gu et al., 2009; Thompson et al., 2009; Scaramuzzino et al., 2015; Todd et al., 2015) and can both enhance and reduce toxicity in SCA3 depending on the site of modification (Fei et al., 2007; Tao et al., 2008; Mueller et al., 2009; Matos et al., 2016). In association with the aggregation-prone family of polyQ disorders, the role of ubiquitination is typically that of alleviating toxicity. RNF4-mediated ubiquitination increases degradation and reduces the toxicity of expanded ataxin-1 and ataxin-7 (Lebre et al., 2001; Guo et al., 2014; Wan et al., 2018), and ubiquitination events can increase the degradation of expanded huntingtin and ataxin-3 (Matsumoto et al., 2004; Steffan et al., 2004; Jana et al., 2005; Miller et al., 2005; Winborn et al., 2008; Todi et al., 2009, 2010; Tsou et al., 2013, 2015b; Sutton et al., 2017).

### Proteolytic processing and sub-cellular localization

Nuclear localization and caspase cleavage are featured in various polyQ disorders. Five of the 9 polyQ disorders possess one or more documented NLS that impact pathogenesis: DRPLA, SBMA, SCA1, SCA6, and SCA7 (Klement et al., 1998; Chen et al., 2003; Nucifora et al., 2003; Young et al., 2007; Lai et al., 2011; Du and Gomez, 2018). Ataxin-3 may also possess one, but its mutation has not had distinct effects on its localization (Blount et al., 2014; Ristic et al., 2018). In each case, the presence of an intact NLS enhances nuclear retention and toxicity, and mutations to render the respective NLS non-functional alleviate toxicity. Additionally, the DRPLA and SCA7 disease proteins also possess export sites whose mutations increase toxicity, indicating that cytoplasmic export is protective (Nucifora et al., 2003; Young et al., 2007; Du and Gomez, 2018). Similar to the NLS, cleavage sites tend to enhance toxicity. Possession of proteolytic sites and the formation of polyQ-containing protein fragments enhance toxicity in DRPLA, HD, SCA3, SCA7, and SBMA (Miyashita et al., 1997; Ellerby et al., 1999a; Kim et al., 2001; Lin et al., 2001; Gafni and Ellerby, 2002; Lunkes et al., 2002; Wellington et al., 2002; LaFevre-Bernt and Ellerby, 2003; Nucifora et al., 2003; Berke et al., 2004; Luo et al., 2005; Graham et al., 2006; Schilling et al., 2006; Truant et al., 2007; Young et al., 2007; Jung et al., 2009; Suzuki et al., 2010; Costa and Paulson, 2012; Hubener et al., 2013; Harmuth et al., 2018).

### Protein-protein interactions

Along with the posttranslational modifications, localization signals, and cleavage sites, polyQ disease proteins also share protein interactions that influence their toxicity. These interactions involve non-polyQ overlaps in each protein, as well as commonalities in the types of interactions that take place through their shared polyQ. One example is TAF_II_130, a coactivator in the cAMP-responsive element-binding protein (CREB)-dependent transcriptional activation (Shimohata et al., 2000). PolyQ-containing proteins preferentially bind TAF_II_130 and suppress its activation; data from yeast two-hybrid models indicate that the interaction between TAF_II_130 (also known as TAF4, or TATA-box-binding protein associated factor 4) and atrophin-1, huntingtin, ataxin-2, and ataxin-3 is stronger when they possess an expanded polyQ. This suppression of activation can be overcome by overexpressing either TAF_II_130 or CREB (Shimohata et al., 2000; Nucifora et al., 2001). The polyQ-mediated interference of CREB-dependent transcriptional activity suggests a shared toxic gain-of-function that contributes to pathogenesis in several polyQ diseases.

In another potentially shared toxic gain-of-function, proteins with polyQ expansions affect cellular activities that are dependent on kinesin and dynein, particularly fast axonal transport (Szebenyi et al., 2003). Models of HD and SBMA indicate that polyQ-expanded proteins compromise fast axonal transport based on characteristic inhibition of anterograde and retrograde flow in isolated axoplasm and cultured cells. This mode of dysfunction may apply beyond HD and SBMA (Szebenyi et al., 2003).

The DUB, USP7 is an additional factor with purported associations with multiple polyQ disease proteins. Discussed earlier in the section on SBMA, USP7 preferentially interacts with polyQ-expanded AR in a mechanism that increases its aggregation. This interaction is likely not exclusive to AR and may target polyQ proteins more generally: USP7 knockdown suppresses degeneration in a *Drosophila* model of SCA3. Differential interactions with USP7 have also been reported between wild-type and expanded ataxin-1 in SCA1 mammalian cell models, and with mutated huntingtin (Pluciennik et al., 2021). These results suggest that USP7 plays a functional role in polyQ pathophysiology in general.

Overlaps in polyQ disease proteins’ interactomes do not always produce the same outcome. While overexpression of the UPS shuttle protein, Ubiquilin-2 in cells reduces the amount of aggregated mutant huntingtin, it induces the accumulation of cytoplasmic ataxin-3 aggregates in the neurons of SCA3 model mice (Gerson et al., 2020). These results indicate selective action of Ubiquilin-2 on different polyQ proteins. The Ubiquilin-2 example accentuates the importance of protein context in pathogenesis—polyQ expansions alone are not sufficient in promoting Ubiquilin-2-mediated clearance of different polyQ proteins; additional domains are required for its outcomes (Gerson et al., 2020).

PolyQ disease protein interactions with important outcomes for pathogenesis can also involve trans-polyQ binding. Some polyQ proteins act on one another. Ataxin-2 accumulates in cells with ataxin-1 nuclear inclusions in SCA1 fly models and human SCA1 neurons (Al-Ramahi et al., 2007). Studies in SCA1 flies found that overexpression and knockdown of ataxin-2 enhances and reduces ataxin-1-dependent toxicity, respectively (Al-Ramahi et al., 2007). The SCA3 protein, ataxin-3, is also found in cellular aggregates from various polyQ diseases; the physiological implications of this localization are unclear. Based on a study with an HD mouse model, *atxn3*-null mice fared similarly to *atxn3*-containing counterparts, suggesting that ataxin-3 is not a key player in polyQ pathogenesis more generally (Zeng et al., 2013).

Splicing factors are another common link among some polyQ diseases. One example is the mRNA splicing factor, RNA-binding Fox-1 homolog 2 (FOX-2) (Lim et al., 2006; Welzel et al., 2012). FOX-2 interacts with ataxin-1 and FOX-2 activity is reduced when ataxin-1 localizes to NI. FOX-2 also binds ataxin-2. This interaction impacts the splicing of *atxn2* pre-mRNA, an outcome that is altered by over-expression of pathogenic ataxin-1 (Lim et al., 2006; Welzel et al., 2012). Interactions that impact splicing may influence polyQ pathogenesis more widely because splice variants have been identified in SCA3 (Goto et al., 1997; Bettencourt et al., 2010; Harris et al., 2010; Johnson et al., 2019) and SCA6 (Tsunemi et al., 2008); they also highlight connections among polyQ proteins and their potential to act on one another, also evidenced by the reduction in ataxin-2 levels in SCA3 samples (Nóbrega et al., 2015) and the appearance of the SCA17 protein in the NI of DRPLA, HD, SCA1, SCA2, and SCA3 (van Roon-Mom et al., 2005; Toyoshima and Takahashi, 2018).

Lastly, we comment on polyproline regions in polyQ proteins. As mentioned above, ataxin-7, atrophin-1, and huntingtin contain polyproline regions. In the case of ataxin-7 and huntingtin, these repeats flank the polyQ track; the polyproline repeat in atrophin-1 is separate from the polyQ. To the best of our knowledge, there is no clear evidence that the polyproline regions impact polyQ toxicity in DRPLA, HD and SCA7.

As the list of protein interactions and pathogenic mechanisms grows for each of the polyQ diseases, it will be important to continue identifying commonalities that establish broader and targetable pathways as therapeutic entry points.

## Concluding remarks

The polyQ diseases comprise a single family. Each disease is rooted in the same type of mutation, whose pathogenic effect is tightly influenced by other parts of the host protein. The information summarized here is a primer to the myriad investigations that have helped to advance our understanding of the protein context surrounding the nine polyQ disease proteins. There are numerous studies of toxic effects that are outside the scope and space dedicated to this review, but certainly provide valuable information about contributors to CAG triplet repeat expansion disorders, including repeat-association non-AUG translation and mRNA toxicity (Zu et al., 2011; Cleary et al., 2018; Guo et al., 2022). These and other processes play important roles in establishing the distinct pathogenesis and clinical presentation of each polyQ disease.

The current state of the field has yielded useful information on the types of approaches that can be beneficial in the clinic. These include, but are not limited to, reducing the levels of the insulting protein by targeting its transcript or enhancing its degradation; preventing or reversing polyQ protein aggregation; regulating posttranslational modification sites or protein-protein interactions that are protective in various cell and animal models. Still, therapeutics are presently unavailable, indicating that continued investigation of polyQ diseases and of their protein context is needed to unlock their mysteries and to enable successful interventions.

## Author contributions

SJ, W-LT, and ST conceptualized the review. SJ, W-LT, MP, AH, and ST collected information and prepared and edited the manuscript. SJ and ST wrote the manuscript. W-LT designed the figures. All authors contributed to the article and approved the submitted version.
